# An Inflammation‐Targeting Engineered Probiotic *Escherichia coli* Nissle 1917 with High Anti‐TNF‐α Nanobody Secretion Efficacy Alleviates Ulcerative Colitis

**DOI:** 10.1002/advs.202512360

**Published:** 2025-09-29

**Authors:** Siqi Hua, Kaiqiang Li, Pengyou Shang, Lina Liu, Jiayu Pan, Bo Zhu, Zichun Hua

**Affiliations:** ^1^ School of Biopharmacy China Pharmaceutical University Nanjing 211198 P. R. China; ^2^ State Key Laboratory of Natural Medicines China Pharmaceutical University Nanjing 211198 P. R. China; ^3^ State Key Laboratory of Pharmaceutical Biotechnology School of Life Sciences Nanjing University Nanjing 210023 P. R. China; ^4^ Peter Gorer Department of Immunobiology School of Immunology and Microbial Sciences King's College London London SE1 9RT UK; ^5^ Johns Hopkins Bloomberg School of Public Health Baltimore MD 21205 USA; ^6^ Faculty of Pharmaceutical Sciences Xinxiang Medical University Xinxiang 453004 P. R. China; ^7^ Changzhou High‐Tech Research Institute of Nanjing University and Jiangsu TargetPharma Laboratories Inc. Changzhou 213164 P. R. China

**Keywords:** annexin A5, anti‐TNF‐α nanobodies, *Escherichia coli* Nissle 1917, inflammatory bowel disease, targeted drug delivery

## Abstract

The probiotic *Escherichia coli* Nissle 1917 (EcN), clinically used for ulcerative colitis (UC) due to its safety and genetic tractability, exhibits limited therapeutic efficacy owing to poor targeting and colonization at inflamed sites. Phosphatidylserine (PS) exposure increases on apoptotic colon epithelial cells during UC progression, suggesting PS‐targeting could enhance EcN localization. Here, EcN is engineered to surface‐display Annexin A5 (ANXA5), a PS‐binding protein, to improve inflammation targeting and colonization. Since TNF‐α drives UC pathogenesis and anti‐TNF‐α biologics face cost and safety limitations, the secretion‐hindering *lpp* gene is concurrently knocked out in ANXA5‐expressing EcN, creating the inflammation‐targeted, secretion‐enhanced EcN*Δlpp*::A5. This base strain is further modified to secrete an anti‐TNF‐α nanobody (aTN), generating EcN*Δlpp*::A5‐aTN. Both engineered strains demonstrate significantly stronger colonic colonization versus wild‐type EcN, effectively attenuating oxidative stress‐mediated epithelial apoptosis, restoring mucosal barriers, improving immune‐microbiota balance, and alleviating murine colitis. EcNΔ*lpp*::A5‐aTN shows superior efficacy to EcNΔ*lpp*::A5. This study develops an engineered EcN system with enhanced targeting, colonization, and secretion. By efficiently delivering anti‐TNF‐α nanobodies, EcNΔ*lpp*::A5‐aTN exhibits strong therapeutic potential for UC, overcoming limitations that hinder the clinical application of wild‐type EcN.

## Graphical abstract

Ulcerative colitis therapy requires precise drug targeting. This study engineers a probiotic *E. coli* Nissle 1917 to surface‐display ANXA5, enabling specific binding to phosphatidylserine (PS) exposed on inflamed colonic epithelium for enhanced targeting and colonization. The strain is further modified to secrete anti‐TNF‐α nanobodies locally, demonstrating superior efficacy over the wild‐type probiotic and infliximab in alleviating murine colitis.

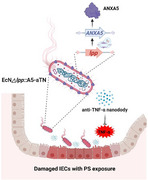

## 1 Introduction

The probiotic *Escherichia coli* Nissle 1917 (EcN) has been approved in multiple countries for the early treatment of ulcerative colitis (UC).^[^ ^1^ ^]^ Compared with traditional drugs, EcN offers advantages such as low production cost, in vivo proliferative capacity, and ease of genetic modification.^[^ ^2^, ^3^ ^]^ However, its clinical efficacy is limited by its poor ability to colonize the intestine.^[^ ^4^, ^5^ ^]^ Previous studies have enhanced the colonization capacity of EcN through chemical modification or nanoparticle encapsulation to improve therapeutic efficacy.^[^ ^6^, ^7^ ^]^ Nevertheless, as bacteria proliferate, these modified compounds or nanomaterials gradually become diluted and cannot exert long‐term effects. We propose leveraging genetic modifiability of EcN to perform multifunctional directed modifications targeting promising and rationally selected biological mechanisms of inflammatory bowel disease, thereby enhancing its therapeutic effect on colitis.

Apoptosis of enterocytes plays a critical role in the occurrence and development of UC by damaging the intestinal epithelial mucosal barrier.^[^ ^8^, ^9^ ^]^ Phosphatidylserine (PS) externalization is a recognized early marker of apoptosis, and Annexin A5 (ANXA5) is commonly used to detect apoptosis in vitro due to its specific binding to PS.^[^ ^10^, ^11^ ^]^ In this study, we hypothesized that genetically engineering EcN to display ANX5A on its surface would target early apoptotic enterocytes and enhance EcN colonization at inflamed colonic sites, thereby improving therapeutic efficacy.

Probiotics generally have limited efficacy for patients with moderate‐to‐severe UC.^[^ ^12^, ^13^ ^]^ Due to the central role of TNF‐α in mediating inflammatory cascade reactions, monoclonal antibodies targeting TNF‐α, such as infliximab, are used to treat advanced UC.^[^ ^14^, ^15^ ^]^ However, clinical practice reveals that the application of expensive infliximab faces multiple challenges, including increased risk of tuberculosis infection from systemic administration, a 30%–50% clinical recurrence rate within 1 year after discontinuation, and ≈10.5% of patients experiencing allergic reactions such as dyspnea or rash.^[^ ^16^ ^]^ Based on the idea of using ANXA5 to enhance intestinal colonization, we hypothesized that further genetic modification of EcN, including knocking out the *lpp* gene, which hinders protein secretion, and engineering the secretion of anti‐TNF‐α nanobodies (aTN), could enhance its therapeutic effect on colitis. In this study, we constructed a bifunctional engineered bacterium, EcN*Δlpp*::A5‐aTN, that displays the PS‐binding protein, ANX5A, on its surface and secretes the anti‐TNF‐α nanobody with high efficacy. We systematically evaluated its therapeutic efficacy in a UC mouse model.

## 2 Results

### 2.1 Construction of EcN*Δlpp*::A5 Engineered Bacteria based on the Increase of Colonic Phosphatidylserine in Inflammatory State

**Figure 1:**
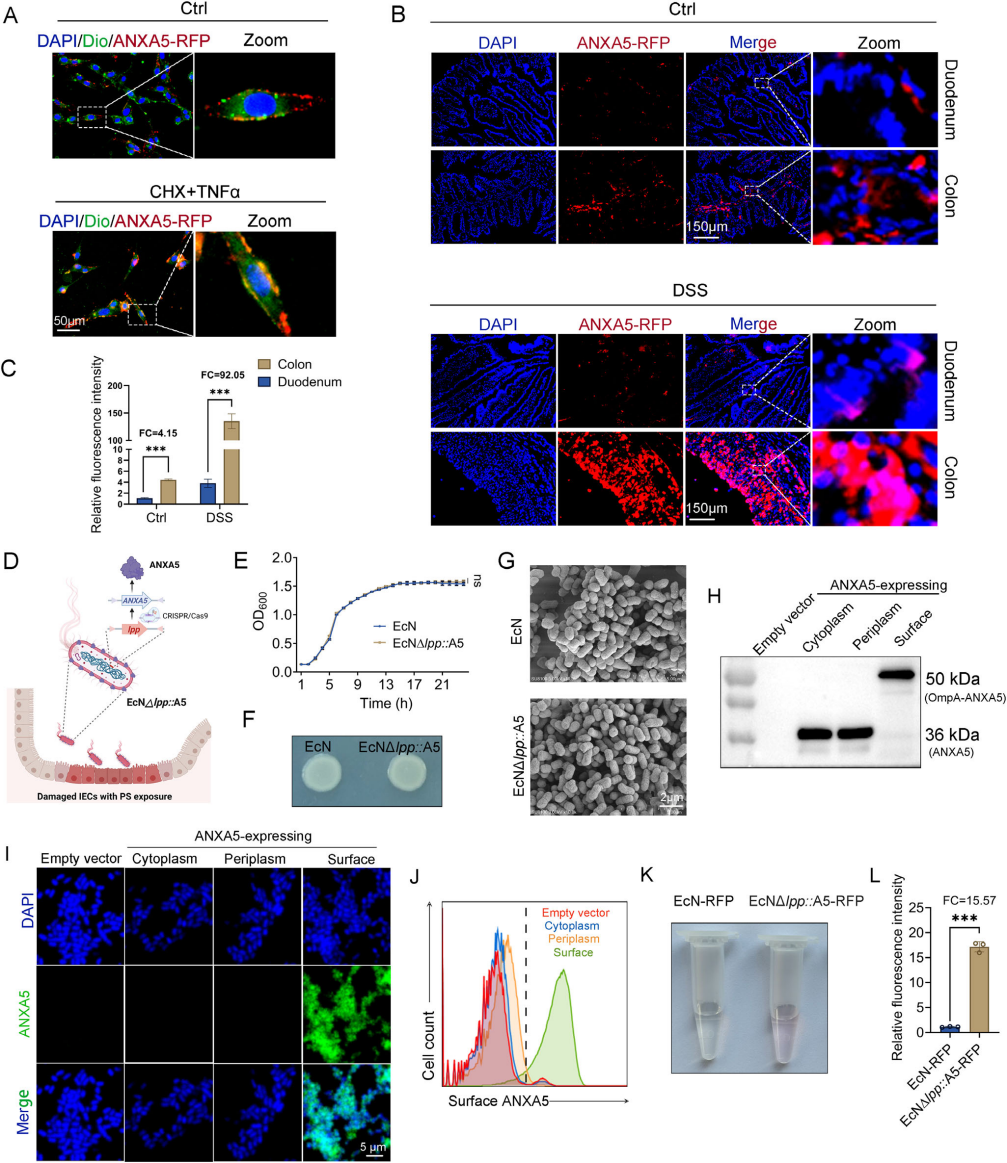
Construction of inflammation‐targeting and secretion‐enhanced EcN*Δlpp*::A5 engineered bacteria. A) CT26 colon epithelial cells were treated with 25 µg mL^−1^ CHX and 20 ng mL^−1^ TNF‐α for 24 h to induce apoptosis. Then, the cells were incubated with ANXA5‐RFP at room temperature for 30 min. The nuclei were stained with DAPI (blue) and the membranes with Dio (green) to visualize ANXA5‐RFP binding to the apoptotic CT26 cells versus the controls. B,C) Mice with DSS‐induced colitis and controls received 20 mg kg^−1^ of ANXA5‐RFP via tail vein injection. After 30 min, frozen sections of the duodenum and colon were analyzed for ANXA5‐RFP accumulation (*n* = 3). D) Schematic of EcN*Δlpp*::A5 construction. E) Bacterial growth curves of EcN and EcN*Δlpp*::A5 (*n* = 3). F) Colonial morphology of EcN and EcN*Δlpp*::A5. G) SEM of EcN and EcN*Δlpp*::A5. H) Expression status of ANXA5 of different engineered EcN strains by western blotting. Empty vector: strain not carrying ANXA5. Cytoplasm: ANXA5 is expressed in the cytoplasm of EcN‐ANXA5. Periplasm: ANXA5 is expressed in the periplasm of EcN‐pelB‐ANXA5. Surface: ANXA5 is expressed in the outer membrane surface of EcNΔlpp::A5 (*n* = 3). I,J) Immunofluorescence I) and flow cytometry J) detection of surface‐displayed ANXA5 on EcN*Δlpp*::A5 (n = 3). K,L) RFP secretion levels (*n* = 3). Data are presented as mean ± SD. Statistical analyses were performed using unpaired two‐tailed t‐test (two‐group comparisons), or two‐way ANOVA with Sidak's test (two independent variables). ***p < 0.001, ns: not significant.

Intestinal epithelial cell death, which is characterized by the exposure of PS, initiates and exacerbates colitis.^[^ ^17^, ^18^ ^]^ Therefore, we hypothesized that expressing the PS‐binding protein ANXA5 on EcN would enhance its colonization of the gut. Immunofluorescence confirmed that combined CHX and TNF‐α treatment induced CT26 cell death and significantly promoted Annexin A5‐red fluorescent protein (ANXA5‐RFP) binding to cell membranes (**Figure** **1**A). Following the intravenous injection of 20 mg kg^−1^ of ANXA5‐RFP protein into DSS‐induced UC mice and controls, we observed greater ANXA5‐RFP accumulation in the colon than in the duodenum. DSS modeling further increased intestinal accumulation (Figure 1B,C). These results suggest that displaying ANXA5 on EcN could promote probiotic enrichment at colonic lesion sites.

Subsequently, we constructed EcN*Δlpp*::A5, which is based on EcN and aims to enhance gut colonization by displaying the ANXA5 on the cell surface and increasing target protein secretion by knocking out the lipoprotein *lpp* gene (Figure 1D). OD_600_ measurements showed no significant difference in proliferation rates between EcN*Δlpp*::A5 and wild‐type EcN (Figure 1E). Plate culture experiments revealed no differences in colony size or morphology (Figure 1F). Scanning electron microscopy (SEM) revealed similar bacterial size and morphology (Figure 1G). The successful integration of *ANXA5* into the EcN*Δlpp*::A5 genome was confirmed by PCR using genomic DNA as a template (Figure S1, Supporting Information).

To confirm the surface display of ANXA5 rather than its localization in other cellular compartments, we constructed two control strains: EcN‐ANXA5, with cytoplasmic expression of ANXA5, and EcN‐pelB‐ANXA5, in which ANXA5 was targeted to the periplasmic space via an N‐terminal pelB signal sequence. Western blot analysis confirmed soluble expression of ANXA5 in all constructed strains (Figure 1H). We then performed immunofluorescence staining (Figure 1I) and flow cytometry (Figure 1J) on intact bacterial cells without permeabilization to evaluate surface accessibility of ANXA5. Since only surface‐exposed ANXA5 would be accessible to its antibody under non‐permeabilizing conditions, specific binding is indicative of outer membrane localization. As expected, strong fluorescence signals were detected specifically in EcN*Δlpp*::A5, whereas no significant binding was observed in the control strains (Figure 1I,J). These results demonstrate that ANXA5 is successfully displayed on the outer surface of EcN*Δlpp*::A5. We used engineered bacteria carrying red fluorescent protein (RFP) to assess protein secretion efficiency and observed a significant increase in red fluorescence intensity in the supernatant of EcN*Δlpp*::A5 cultures (Figure 1K,L).

These results demonstrate that ANXA5 was successfully displayed on EcN*Δlpp*::A5 and that the *lpp* knockout significantly enhances the efficiency of secreting exogenous proteins.

### 2.2 Enhanced Gut Colonization Capacity of EcN*Δlpp*::A5

**Figure 2:**
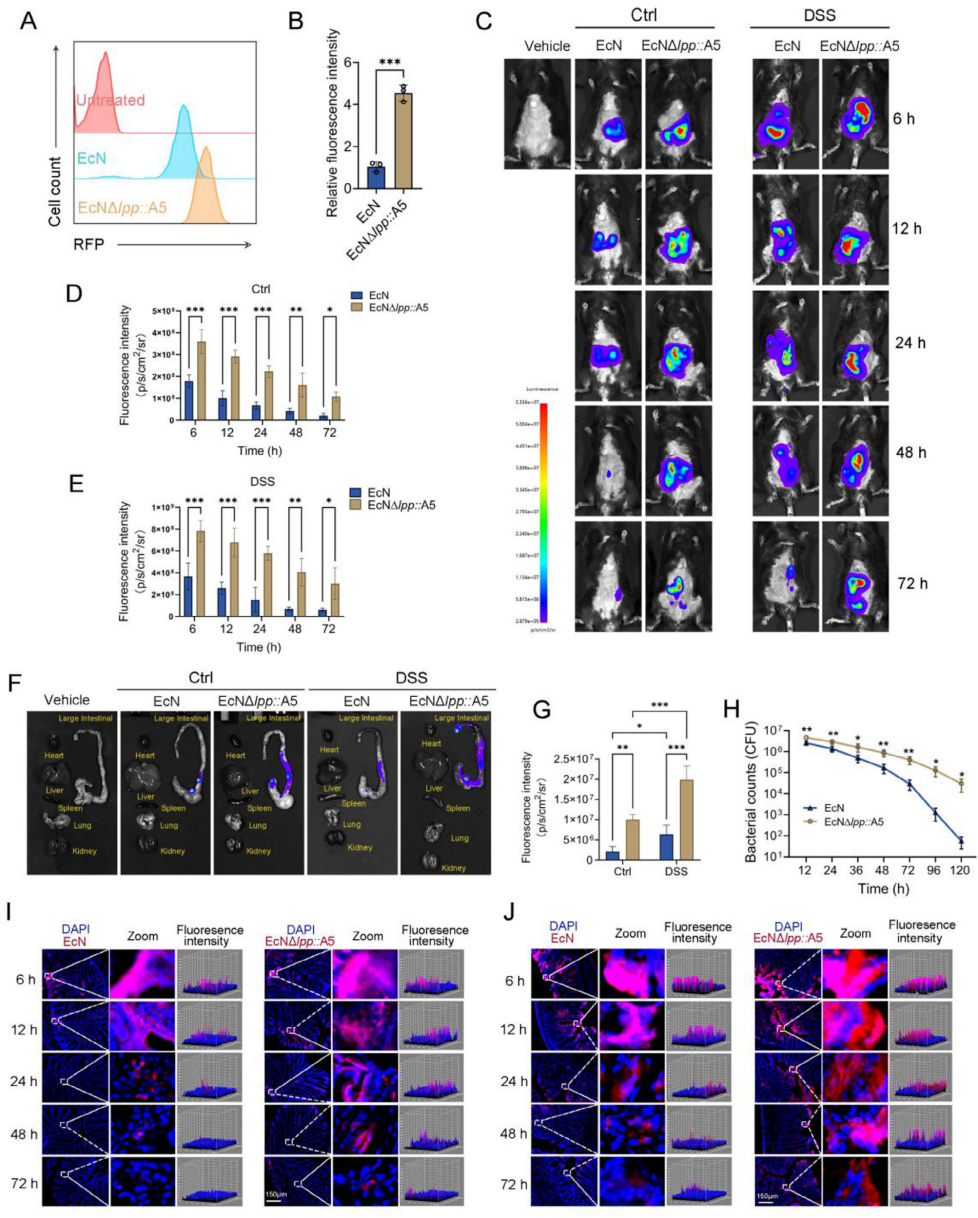
Evaluation of the intestinal colonization capacity of EcN*Δlpp*::A5 engineered bacteria. A,B) After inducing apoptosis in CT26 colon epithelial cells, RFP‐labeled EcN and EcN*Δlpp*::A5 were added to the culture system at a MOI of 10:1 and incubated for 1 h. Non‐specifically bound bacteria were removed by washing with PBS, and flow cytometry was used to compare the binding of both bacteria to apoptotic CT26 cells. (*n* = 3) C–E) The LuxCDABE bioluminescence system was introduced into EcN and EcN*Δlpp*::A5, respectively. DSS‐induced colitis mice and control mice were orally administered with 1 × 10⁹ CFU of each bacterium. The in vivo imaging system was used to compare colon colonization capacity at the time points of 6–72 h (*n* = 3). F,G) 72 h after gavage, the mice were euthanized and dissected to compare bacterial colonization in the heart, liver, spleen, lungs, kidneys, and colon tissues at the organ level. (*n* = 3) H) DSS‐induced colitis mice and control mice were orally administered 1 × 10⁹ CFU of wild‐type EcN and EcN*Δlpp*::A5, respectively. At the time points of 12–120 h, colon tissues were collected for homogenization, and the capacity for bacterial colonization was dynamically compared using the plate‐spreading method (*n* = 3). I,J) Control mice and DSS‐induced colitis mice were orally administered 1×10⁹ CFU of EcN‐RFP and EcN*Δlpp*::A5‐RFP, respectively. At the time points of 6–72 h, the mice were euthanized, and frozen sections were prepared to observe bacterial colonization in the colon tissues under a fluorescence microscope (*n* = 3). Data are presented as mean ± SD. Statistical analyses were performed using unpaired two‐tailed t‐test (two‐group comparisons), or two‐way ANOVA with Sidak's test (two independent variables). *p < 0.05, **p < 0.01, ***p < 0.001.

EcN*Δlpp*::A5 expressing red fluorescent protein (EcN*Δlpp*::A5‐RFP) and EcN expressing red fluorescent protein (EcN‐RFP) were co‐cultured with apoptotic CT26 cells that were induced by a combined treatment of CHX and TNF‐α. EcN*Δlpp*::A5 exhibited a significantly higher binding capacity to CT26 cells with phosphatidylserine exposure than EcN did (**Figure** **2**A,B). We introduced the LuxCDABE bioluminescence system into both EcN*Δlpp*::A5 and EcN. Control and DSS‐induced colitis mice received simultaneous oral gavage of 1 × 10⁹ CFU doses of both EcN and EcN*Δlpp*::A5 strains. In vivo imaging at 6, 12, 24, 48, and 72 h post‐gavage revealed that EcN*Δlpp*::A5 exhibited stronger intestinal colonization than EcN not only in the colitis group but also in healthy controls (Figure 2C–E). Furthermore, both strains colonized the intestines of UC mice more effectively than those of control mice (Figure S2, Supporting Information). Organ dissection 72 h post‐gavage revealed that both strains primarily colonized the large intestine with no bacterial colonization in the heart, liver, spleen, lungs, or kidneys (Figure 2F,G), indicating high safety of EcN*Δlpp*::A5. DSS‐induced UC mice were gavaged with EcN*Δlpp*::A5 or EcN. Dynamic plate count analysis of intestinal colonization from 12 to 120 h demonstrated that EcN*Δlpp*::A5 exhibited significantly higher colonization levels at every time point compared to the wild‐type strain (Figure 2H). Notably, by 120 h, wild‐type EcN was nearly cleared, whereas EcN*Δlpp*::A5 maintained substantial colonization (Figure 2H). RFP‐labeled EcN*Δlpp*::A5 and EcN were administered to DSS‐induced UC mice and controls via gavage. Cryosectioning combined with fluorescence microscopy at 6–72 h confirmed colonization patterns consistent with in vivo imaging (Figure 2I,J).

### 2.3 Oral EcN*Δlpp*::A5‐aTN Demonstrates Excellent Safety in mice

**Figure 3:**
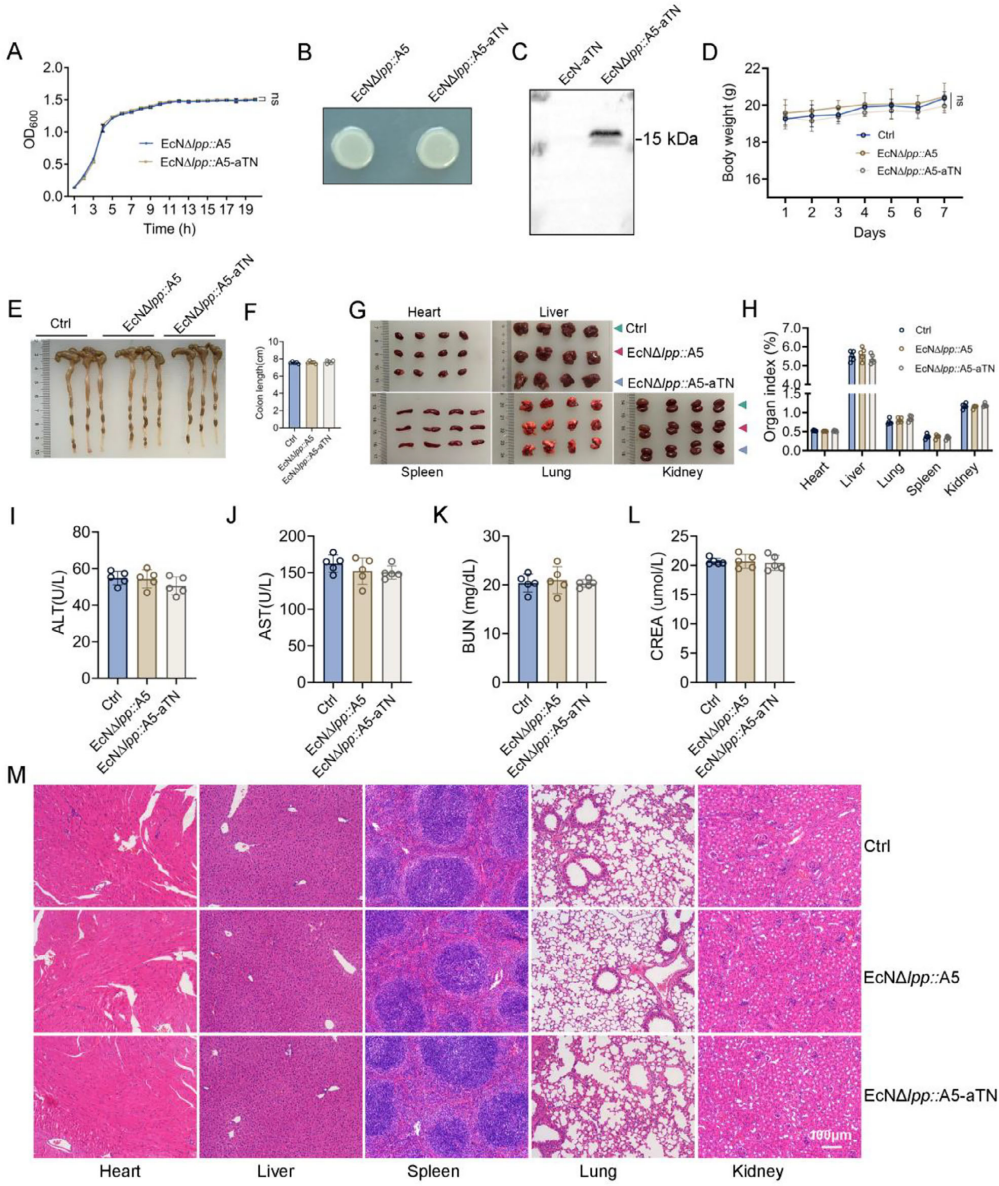
Construction and safety evaluation of EcN*Δlpp*::A5‐aTN engineered bacteria. A) Bacteria growth curves of EcN*Δlpp*::A5 and EcN*Δlpp*::A5‐aTN (*n* = 3). B) Colonial morphology of EcN*Δlpp*::A5 and EcN*Δlpp*::A5‐aTN. C) Under the same culture conditions, the culture supernatants of EcN‐aTN and EcN*Δlpp*::A5‐aTN were collected, and aTN secretion levels were compared by western blotting (*n* = 3). Healthy mice received an oral gavage of 1 × 10⁹ CFU of either EcN*Δlpp*::A5 or EcN*Δlpp*::A5‐aTN for seven days (*n* = 5). D) Mouse body weight changes. E,F) On day 7, the mice were euthanized, and the colon length was measured. G) Heart, liver, spleen, lung and kidney tissues were photographed. H) Organ indexes were calculated as organ weight/body weight×100. I–L) Mouse serum was isolated to detect liver injury markers (ALT and AST) and kidney injury markers (BUN and CREA). M) H&E staining was used to evaluate tissue damage in heart, liver, spleen, lung and kidney. Statistical analyses were performed using one‐way ANOVA with Tukey's test (≥3 groups), or two‐way ANOVA with Sidak's test (two independent variables). ns: not significant.

TNF‐α is a key driver of inflammation in UC.^[^ ^19^, ^20^ ^]^ After confirming the enhanced colonization and protein secretion of EcN*Δlpp*::A5, we transformed this strain with a plasmid encoding an aTN to create EcN*Δlpp*::A5‐aTN. Growth curves (**Figure** **3**A) and colony morphology (Figure 3B) revealed no differences compared to the EcN*Δlpp*::A5 control. A western blot of bacterial supernatants demonstrated significantly higher aTN secretion efficiency in EcN*Δlpp*::A5‐aTN than in EcN‐aTN (Figure 3C). When EcN*Δlpp*::A5‐aTN was cultured in Luria‐Bertani (LB) medium to an OD_600_ of 0.9, corresponding to a bacterial concentration of ≈1 × 10^9^ CFU mL^−1^, the concentration of aTN in the supernatant was determined by ELISA to be 1332.79±172.28 ng mL^−1^ (Figure S3, Supporting Information). In both simulated gastric fluid and bile salts, the survival rates of EcN*Δlpp*::A5‐aTN, EcN*Δlpp*::A5, and EcN showed no significant differences, suggesting that the genetic modifications did not compromise the resistance of these bacteria to the gastrointestinal environment (Figure S4, Supporting Information). As a commercially available drug in Germany and other European countries, the safety of wild‐type EcN is widely recognized. Consistently, we found that oral gavage of 1 × 10⁹ CFU EcN had no obvious toxic effects on mice (Figure S5, Supporting Information). We then proceeded to further evaluate the in vivo safety of our engineered strains, EcN*Δlpp*::A5‐aTN and EcN*Δlpp*::A5 (Figure 3D–M). Healthy mice gavaged with 1 × 10⁹ CFU of either EcN*Δlpp*::A5‐aTN or EcN*Δlpp*::A5 showed no differences in colon length, body weight (Figure 3D–F), organ morphology, or organ index compared to controls (Figure 3G,H). Serum markers remained within normal ranges, including liver injury markers ALT (Figure 3I) and AST (Figure 3J) and kidney injury markers BUN (Figure 3K) and CREA (Figure 3L). H&E staining revealed no significant pathological damage or inflammatory cell infiltration in heart, liver, spleen, lung, or kidney tissues (Figure 3M). These results demonstrate the excellent safety of the oral EcN*Δlpp*::A5‐aTN strain.

### 2.4 EcN*Δlpp*::A5‐aTN Significantly Alleviates Murine Colitis

**Figure 4:**
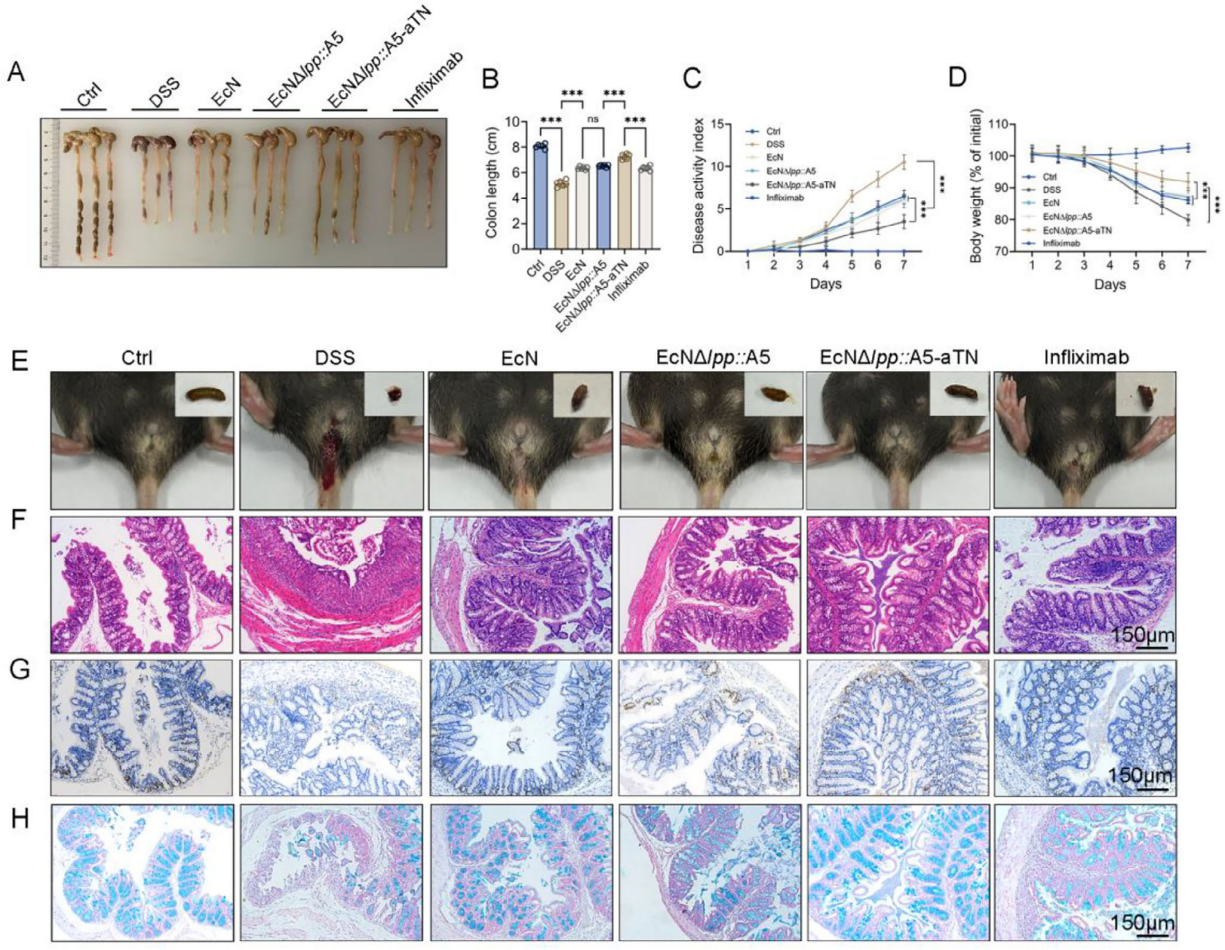
Evaluation of therapeutic effects of EcN*Δlpp*::A5‐aTN on mouse UC model. The UC mouse model was established using 3.5% DSS as the sole source of drinking water. The treatment groups received an oral gavage of 1 × 10⁹ CFU of EcN, EcN*Δlpp*::A5, or EcN*Δlpp*::A5‐aTN daily. DSS colitis mice that received an equal volume of PBS served as the model control group (*n* = 6). Non‐modeled mice served as the healthy control group. DSS‐treated mice that received 10 mg kg^−1^ infliximab via intraperitoneal injection on days 3 and 5 served as the positive control group. On day 7 of modeling, the mice were euthanized to compare the therapeutic effects. A,B) Colon length comparison among different groups (*n* = 6). A representative image from each group is shown. C) Changes in DAI over time (n = 6). D) Body weight changes over time (*n* = 6). E) Mouse stool morphology and anal inflammatory bleeding. F) H&E staining to evaluate colon damage. G) Ki67 immunohistochemistry to evaluate intestinal epithelial cell proliferation. H) Alcian blue staining to evaluate goblet cell mucus secretion. Data are presented as mean ± SD. Statistical analyses were performed using one‐way ANOVA with Tukey's test (≥3 groups), or two‐way ANOVA with Sidak's test (two independent variables). ***p < 0.001, ns: not significant.

In DSS‐induced UC model with infliximab as the positive control, EcN*Δlpp*::A5‐aTN outperformed EcN*Δlpp*::A5 and infliximab. UC mice treated with EcN*Δlpp*::A5‐aTN exhibited greater colon length preservation (**Figure** **4**A,B), lower disease activity index (DAI) scores (Figure 4C), improved weight recovery (Figure 4D), normalized stool consistency, and reduced intestinal bleeding (Figure 4E). The therapeutic efficacy of EcN*Δlpp*::A5‐aTN notably surpassed that of infliximab (Figure 4A–E). H&E staining revealed improved colonic histology, including realigned epithelium and intact crypts (Figure 4F). Ki67 staining revealed increased epithelial proliferation (Figure 4G), which facilitates barrier repair. Alcian Blue staining confirmed increased mucus secretion (Figure 4H). These results demonstrate the significant therapeutic effects of EcN*Δlpp*::A5‐aTN.

### 2.5 EcN*Δlpp*::A5‐aTN Suppresses Epithelial Oxidative Stress and Apoptosis

**Figure 5:**
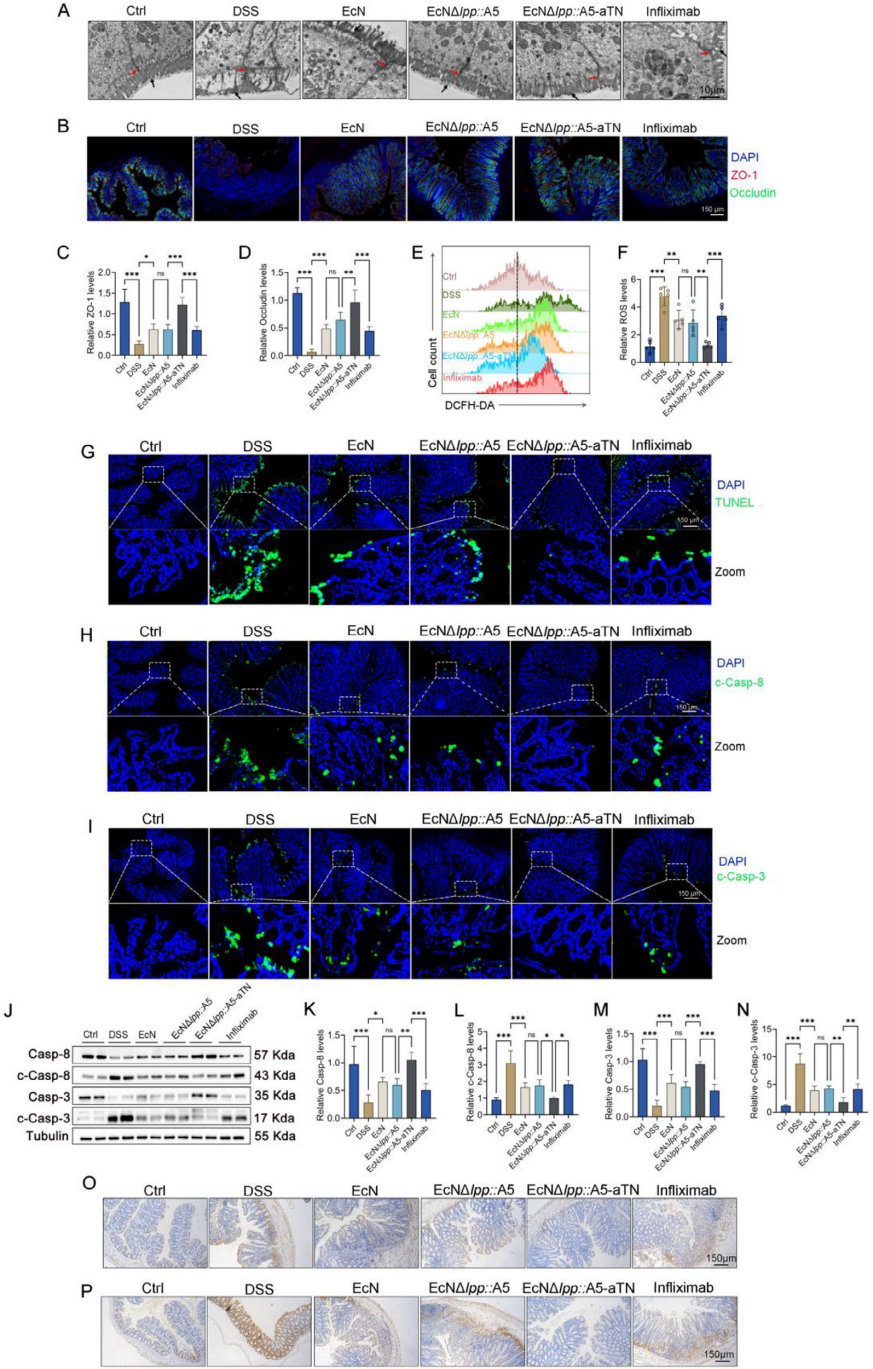
Inhibitory effects of EcN*Δlpp*::A5‐aTN on ROS/JNK/p38‐mediated intestinal epithelial cell apoptosis and promotion of intestinal mucosal tight junction recovery in DSS colitis mice. A) TEM observation of colon tissue tight junctions (red arrows) and villi (black arrows). B–D) Immunofluorescence observation of the expression of the tight junction proteins ZO‐1 and Occludin in colon tissues (*n* = 3). E,F) Primary colon epithelial cells were isolated, and DCFH‐DA staining was used to compare ROS levels (*n* = 5). G) TUNEL staining was used to compare apoptosis in colon tissues (*n* = 3). H) Immunofluorescence to compare cleaved caspase‐8 expression in colon tissues (*n* = 3). I) Immunofluorescence to compare cleaved caspase‐3 expression in colon tissues (*n* = 3). J–N) Western blot analysis of the expression levels of the apoptosis markers caspase‐8, cleaved caspase‐8, caspase‐3 and cleaved caspase‐3 in colon tissues (*n* = 3). O,P) Immunohistochemistry was used to compare p‐JNK and p‐p38 levels in colon tissues (*n* = 3). Data are presented as mean ± SD. Statistical analyses were performed using one‐way ANOVA with Tukey's test. *p < 0.05, **p < 0.01, ***p < 0.001, ns: not significant.

Transmission electron microscopy (TEM) analysis revealed severe structural damage in the colonic epithelium of the DSS‐induced colitis model, characterized by disrupted tight junctions, blurred cellular outlines, and shortened villi. Treatment with EcN*Δlpp*::A5 promoted the restoration of tight junction integrity and improved villus morphology (**Figure** **5**A). Immunofluorescence confirmed the superior restoration of ZO‐1 and Occludin by EcN*Δlpp*::A5‐aTN than EcN*Δlpp*::A5 and infliximab (Figure 5B–D). DCFH‐DA staining revealed elevated ROS in DSS colitis epithelial cells (Figure 5E,F). Mechanistically, we found that consistent with its therapeutic effect, EcN*Δlpp*::A5‐aTN had a stronger downregulatory effect on ROS in intestinal epithelial cells than EcN*Δlpp*::A5 and infliximab (Figure 5E,F). TUNEL staining indicated that the inhibitory effect of EcN*Δlpp*::A5‐aTN on intestinal epithelial cell apoptosis was stronger than that of EcN*Δlpp*::A5 and infliximab (Figure 5G). Immunofluorescence revealed increased extrinsic apoptosis (higher levels of cleaved caspase‐3 and ‐8) in colitis, which was significantly reduced by EcN*Δlpp*::A5‐aTN treatment, and this effect was superior to that of EcN*Δlpp*::A5 and infliximab (Figure 5H,I). Western blot confirmed these findings (Figure 5J–N). IHC showed that EcN*Δlpp*::A5‐aTN had a stronger downregulatory effect on the phosphorylation levels of ROS downstream targets JNK (Figure 5O; Figure S5, Supporting Information) and p38 (Figure 5P; Figure S5, Supporting Information) than EcN*Δlpp*::A5 and infliximab. Taken together, these results demonstrate that EcN*Δlpp*::A5‐aTN alleviates colitis by inhibiting ROS/JNK/p38/Caspase‐8/Caspase‐3‐mediated apoptosis in intestinal epithelial cells, and its effect is superior to both infliximab and the control strain EcN*Δlpp*::A5.

### 2.6 EcN*Δlpp*::A5‐aTN Modulates Intestinal Immune Microenvironment

**Figure 6:**
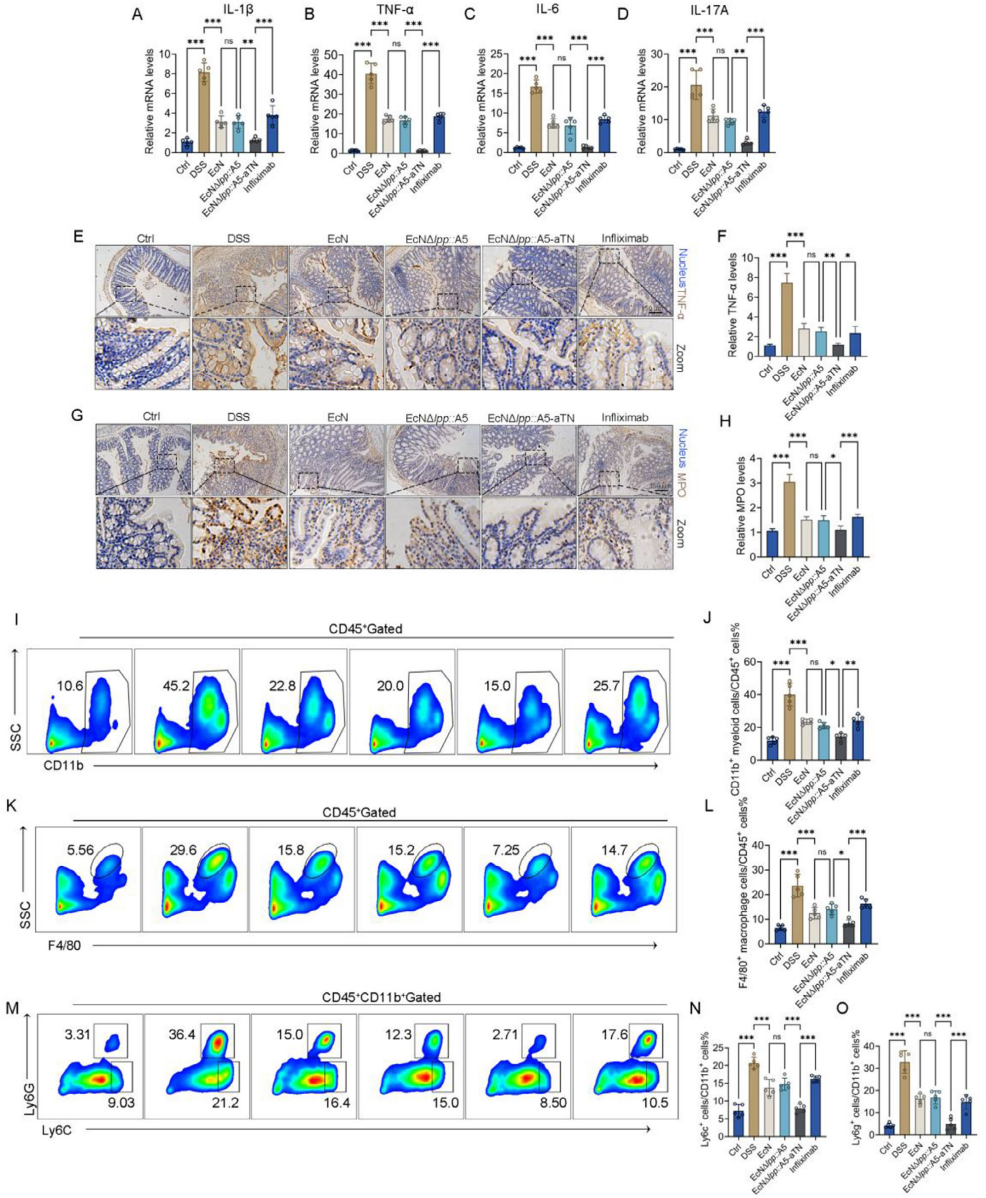
Effects of EcN*Δlpp*::A5‐aTN on the intestinal immune microenvironment in mice with DSS colitis. A–D) qPCR detection of inflammatory cytokine mRNA expression levels in colon tissues (*n* = 5). E,F) Immunohistochemistry to compare TNF‐α expression in colon tissues (*n* = 3). G,H) Immunohistochemistry to compare MPO expression in colon tissues (n = 3). Colonic lamina propria immune cells were isolated and analyzed by flow cytometry (*n* = 5): I,J) CD45^+^CD11b^+^ myeloid cells; K,L) F4/80^+^ macrophages; M,N) Ly6C^+^ monocytes in CD11b^+^ myeloid cells; M,O) Ly6G^+^ neutrophils in CD11b^+^ myeloid cells. Data are presented as mean ± SD. Statistical analyses were performed using one‐way ANOVA with Tukey's test. *p < 0.05, **p < 0.01, ***p < 0.001, ns: not significant.

qPCR revealed that EcN*Δlpp*::A5‐aTN further downregulates colonic IL‐1β (**Figure** **6**A), TNF‐α (Figure 6B), IL‐6 (Figure 6C), and IL‐17A (Figure 6D) mRNA expression compared to EcN*Δlpp*::A5 and infliximab. IHC confirmed a greater reduction in TNF‐α and the neutrophil marker MPO by EcN*Δlpp*::A5‐aTN compared to EcN*Δlpp*::A5 and infliximab (Figure 6E–H). Flow cytometry revealed that DSS‐induced colitis mice increased the number of lamina propria CD11b^+^ myeloid cells (Figure 6I,J), macrophages (Figure 6K,L), Ly6C^+^ monocytes (Figure 6M,N), and Ly6G^+^ neutrophils (Figure 6M,O) within the CD11b^+^ population. Both engineered strains and infliximab reduced these inflammatory cell populations, with EcN*Δlpp*::A5‐aTN exhibiting the strongest effects.

### 2.7 EcN*Δlpp*::A5‐aTN Modulates Gut Microbiota in DSS‐induced Colitis Mice

**Figure 7:**
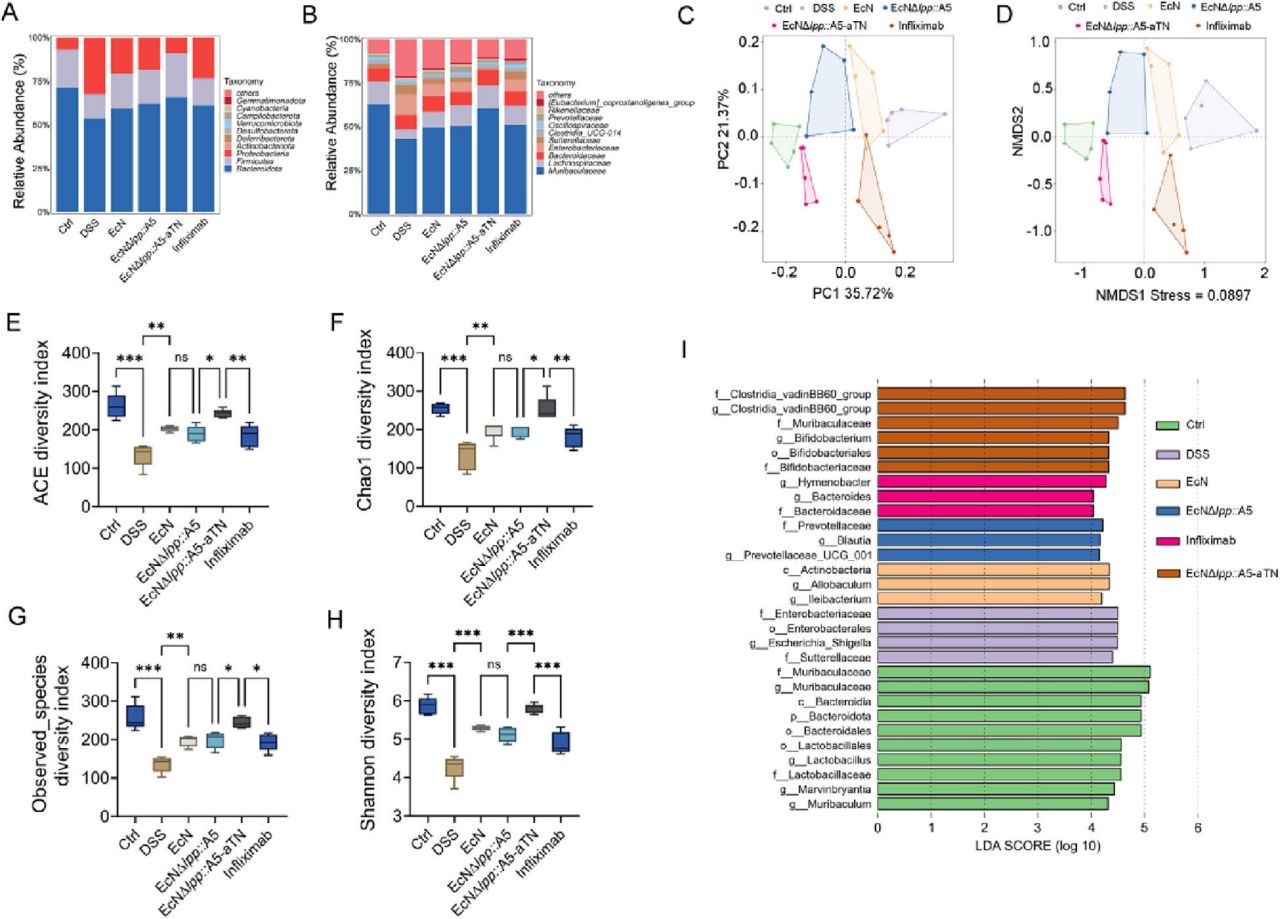
EcN*Δlpp*::A5‐aTN ameliorate gut microbiota dysbiosis in mice with DSS colitis. A) Gut microbiota composition at the phylum level in different groups. B) Gut microbiota composition at the family level in different groups. C) PCoA analysis of each group. D) NMDS analysis of each group. E–H) Changes in ACE, Chao1, observed species and Shannon index in each group. I) Linear discriminant analysis Effect Size (LEfSe) analysis of the microbiota in the different groups. (*n* = 5) Data are presented as mean ± SD. Statistical analyses were performed using one‐way ANOVA with Tukey's test. *p < 0.05, **p < 0.01, ***p < 0.001, ns: not significant.

Dyshomeostasis in gut microbiota play an important role in colitis development.^[^ ^21^ ^]^ To evaluate the impact of EcN*Δlpp*::A5‐aTN on gut microbiota composition, we performed 16S rRNA sequencing on fecal samples from mice with colitis. We observed notable shifts in microbial community structure, visualized through clustered bar charts at both phylum and family levels. At the phylum level (**Figure** **7**A), treatment with EcN*Δlpp*::A5‐aTN significantly increased the relative abundances of Bacteroidota and Firmicutes compared to the DSS group. This shift was largely driven by enrichment of beneficial families such as Muribaculaceae and Lachnospiraceae (Figure 7B). Elevated abundances of Muribaculaceae and Lactobacillaceae are associated with maintenance of gut homeostasis during inflammation. Muribaculaceae contributes to the production of acetate and other short‐chain fatty acids, which lower luminal pH, inhibit pathogenic bacteria, and modulate immune responses.^[^ ^22^ ^]^ Lactobacillaceae enhances epithelial barrier function via lactate and antimicrobial compound secretion, competitively excludes pathogens, elevates anti‐inflammatory IL‐10, and suppresses pro‐inflammatory cytokines.^[^ ^23^, ^24^, ^25^ ^]^ Conversely, EcN*Δlpp*::A5‐aTN administration reduced the abundance of Proteobacteria, including the inflammation‐associated families Sutterellaceae and Enterobacteriaceae (Figure 7B). Enterobacteriaceae, facultative anaerobes that thrive in inflammatory milieus, exacerbate colitis through LPS‐mediated activation of NF‐κB and other pro‐inflammatory pathways, leading to epithelial damage.^[^ ^26^ ^]^ Sutterellaceae, frequently enriched during dysbiosis, may further disrupt microbial balance and perpetuate inflammation.^[^ ^27^ ^]^

Principal coordinate analysis (PCoA) and Nonmetric multidimensional scaling (NMDS) analysis confirmed structural divergence among microbial communities across groups, underscoring the distinctive remodeling effect of EcN*Δlpp*::A5‐aTN on gut microbiota (Figure 7C,D). Alpha diversity analysis revealed a significant reduction in microbial richness and diversity in DSS‐induced colitis mice, as indicated by marked declines in the Ace, Chao1, observed species, and Shannon indices. While treatment with EcN*Δlpp*::A5, EcN, and infliximab each partially restored these metrics, the EcN*Δlpp*::A5‐aTN group exhibited the most pronounced recovery, significantly outperforming all other treatment groups and closely approaching the diversity levels observed in healthy controls (Figure 7E–H). Consistent with these trends, linear discriminant analysis (LDA>4) identified Sutterellaceae and Enterobacteriaceae as significantly enriched in DSS‐control mice (*p* = 0.0029; *p* = 0.0117). In contrast, EcN*Δlpp*::A5‐aTN promoted the growth of Clostridia_vadinBB60_group within the phylum Firmicutes (*p* = 0.0206) and Muribaculaceae within the phylum Bacteroidota (*p* = 0.0011) (Figure 7I).

Overall, these findings demonstrate that EcN*Δlpp*::A5‐aTN effectively restores gut microbial ecology in colitis models, exhibiting superior efficacy compared to both the control strains EcN*Δlpp*::A5 and EcN, as well as the positive control infliximab.

## 3 Discussion

Poor intestinal colonization of the probiotic EcN limits its clinical efficacy in treating colitis.^[^ ^28^, ^29^ ^]^ In this study, we genetically modified an engineered EcN*Δlpp*::A5 bacterium to target inflammation and enhance colonization. We further effectively enhanced its UC therapeutic efficacy by secreting and expressing anti‐TNF‐α nanobodies.

Studies have often reported achieving enhanced bacterial drug targeting and efficacy through encapsulated materials or coupled compounds.^[^ ^5^ ^]^ A highlight of this study is the enhancement of inflammation‐targeting and colonic colonization of EcN via genetic modification. Damaged and dead intestinal epithelial cell PS ectopically translocate from the intracellular membrane to the extracellular membrane,^[^ ^30^ ^]^ making it theoretically feasible to target PS to increase EcN colonization. ANXA5 is a well‐recognized PS‐binding protein, and as such, has been widely used in apoptosis assays in vitro.^[^ ^31^, ^32^ ^]^ By tail vein injection of ANXA5‐RFP protein, we found that colonic PS exposure in healthy mice was 4.15 times higher than that in the duodenum, whereas the level of colonic PS exposure was increased to 92.05 times higher than that in the duodenum under pathologic conditions of enterocolitis, suggesting that PS is more highly exposed at the site of colonic pathology under pathologic conditions and is a potential therapeutic target for colitis.

Utilizing the binding ability of ANXA5 to PS, we displayed ANXA5 on the surface of EcN for the first time, and confirmed by *ex vivo* and in vivo experiments that it can be precisely targeted and colonized on the surface of intestinal epithelial cells that are damaged or dead under inflammatory conditions. In our previous study, we constructed ANXA5 mutants (R25A, K29S, R63S, D68A, E72Q, D144N, E228A, and D303N) of the PS‐affinity binding key amino acids and confirmed that the mutations completely disrupted the ability to bind PS.^[^ ^33^ ^]^ We preliminarily found that EcN bacteria engineered to display this ANXA5 mutant were significantly less capable of treating UC than those displaying the ANXA5 wild type, nearly reverting to the effectiveness of wild‐type EcN (data not shown). This “biological targeting navigation” leverages features of the disease microenvironment, shifting drug delivery from passive diffusion to active recognition. Unlike nanomaterial‐based physical targeting, this approach utilizes the natural colonization advantages of probiotics while avoiding the toxicity of exogenous materials. EcN is a clinically validated, safe probiotic with widely recognized potential in UC therapy.^[^ ^34^, ^35^ ^]^ Clinical trials confirm that its efficacy in treating UC is comparable to that of 5‐aminosalicylic acid, and it is better tolerated.^[^ ^36^ ^]^ EcN can be used for genetic engineering to deliver anti‐inflammatory molecules or modulate gut microenvironments.^[^ ^37^ ^]^ However, despite its genetic tractability, the dense outer membrane of EcN limits the secretion of transmembrane proteins.^[^ ^38^ ^]^ The *lpp*‐encoded lipoprotein connects the outer membrane to the peptidoglycan layer, which restricts the efficiency of secretion.^[^ ^39^ ^]^ Another highlight of this study is in the enhancement of the secretory function of EcN by *lpp* gene knockout. Using RFP as a tracer protein, we demonstrated that *lpp* gene deletion enhanced the secretion efficiency of heterologous proteins in EcN*Δlpp*::A5 by ≈15‐fold, suggesting EcN*Δlpp*::A5 as a promising engineered bacterium with high secretion efficacy

Knocking out *lpp* induces a “leaky phenotype,” which increases outer membrane permeability and promotes direct protein secretion.^[^ ^40^ ^]^ Additionally, as a pathogen‐associated molecular pattern, Lpp may activate TLR2 signaling, and its absence reduces immunogenicity.^[^ ^41^ ^]^ Studies indicate that Lpp increases the immunogenicity and reactivity of *Escherichia coli*. Conversely, *lpp* knockout enhances host tolerance.

As a probiotic, EcN itself offers a certain, though limited, therapeutic effect against ulcerative colitis. This was the initial motivation for genetically engineering it in this study. Although the intestinal colonization ability of EcN*Δlpp*::A5 was significantly stronger than wild‐type EcN, we found that its therapeutic efficacy for colitis was not significantly enhanced. We believe one possible explanation is that at the dose we selected, the therapeutic effect of EcN had already approached its peak, and further increases in colonization offered only limited additional benefit. When the strain was further engineered to secrete aTN, however, it was able to fully leverage its enhanced colonization advantage by directly blocking TNF‐α‐mediated inflammatory signals on top of the inherent therapeutic effects of EcN, thereby significantly improving its efficacy. The significant improvement in colitis treatment efficacy from aTN secretion by EcN*Δlpp*::A5 leads us to believe that EcN*Δlpp*::A5 could have the potential to become a chassis bacterium that can carry a variety of recombinant proteins and antibody drugs due to its superior intestinal targeting and colonization ability, and secretory function. This strategy is promising to address the challenges of high production cost, short half‐life, and high side effects of systemic drug administration of large molecule drugs for colitis treatment.

TNF‐α is a core inflammatory driver and a key therapeutic target for IBD.^[^ ^42^ ^]^ Pathologically, TNF‐α exacerbates inflammation by: 1) activating the NF‐κB pathway, which releases pro‐inflammatory cytokines;^[^ ^43^ ^]^ 2) inducing epithelial apoptosis and disrupting tight junction proteins, which compromises barrier function;^[^ ^43^ ^]^ and 3) promoting inflammatory cells infiltration.^[^ ^44^ ^]^ Clinical studies have shown that TNF‐α levels are elevated in the serum, gut tissues, and feces of patients with active IBD, and these levels correlate with disease severity. Although anti‐TNF‐α monoclonal antibodies can alleviate symptoms, systemic administration has limitations: ≈30% primary non‐response, 20%–40% secondary failure, and risks of infection, hypersensitivity, and anti‐drug antibodies.^[^ ^45^, ^46^ ^]^ Additionally, the large size (≈150 kDa) of conventional antibody drugs hinders mucus penetration, limiting local delivery.^[^ ^47^ ^]^

Clinically, probiotics like EcN are used primarily for early UC intervention, while infliximab treats advanced stages.^[^ ^48^, ^49^ ^]^ EcN*Δlpp*::A5‐aTN combines the advantages of probiotic and anti‐TNF‐α therapies, and it can potentially act across UC progression stages. In this study, localized anti‐TNF‐α nanobody delivery via EcN*Δlpp*::A5‐aTN achieved precise and sustained suppression of TNF‐α at inflamed sites, which could potentially avoid the need for systemic immunosuppression. In this study, we found that EcN*Δlpp*::A5‐aTN can neutralize TNF‐α and cooperatively ameliorate inflammation by inhibiting ROS/JNK/p38/Caspase‐8/Caspase‐3‐mediated apoptosis, upregulating tight junction proteins, and modulating the immune‐microbiome balance in mice with colitis. At least in the DSS‐induced mouse UC model, we found that EcN*Δlpp*::A5‐aTN efficacy was superior to wild type EcN, EcN*Δlpp*::A5, and infliximab. Mechanistically, this may be related to the fact that EcN*Δlpp*::A5‐aTN can be targeted to the site of inflammation and is constantly expressed to secrete aTN. As a probiotic engineered bacterium, the direct regulatory effect of EcN*Δlpp*::A5‐aTN on the balance of intestinal flora may be another advantage over infliximab. Our findings provide a new clue for overcoming the limitations of current anti‐TNF therapy and establishes a theoretical foundation for precision therapy based on host‐microbe interactions through the integration of synthetic biology and targeted delivery.

A recent study demonstrated that EcN, engineered to secrete low‐dose IL‐2 and encapsulated with Eudragit L100‐55 enteric coating, can promote regulatory T cell generation and produce anti‐colitis effects, showcasing a promising strategy to harness the versatility of EcN for reprogramming the gut immune milieu.^[^ ^50^ ^]^ In our study, all four treatments—EcN, EcN*Δlpp*::A5, EcN*Δlpp*::A5‐aTN, and infliximab—significantly lowered the expression of inflammatory cytokines, including IL‐1β, TNF‐α, IL‐6, and IL‐17A, in the inflamed colon. This reduction was accompanied by a corresponding significant decrease in the infiltration of CD11b^+^ myeloid cells, macrophages, monocytes, and neutrophils. While the anti‐inflammatory effects of EcN, EcN*Δlpp*::A5, and infliximab were broadly similar, the impact of EcN*Δlpp*::A5‐aTN on both cytokine expression and inflammatory cell infiltration was markedly superior to that of the other groups. Rapid suppression of myeloid infiltration during acute colitis effectively controls inflammation.^[^ ^51^ ^]^ However, systemic anti‐TNF therapies struggle to sustain efficacy due to their short half‐lives and poor accumulation at the site of inflammation.^[^ ^52^, ^53^ ^]^ Enhanced colonization of EcN*Δlpp*::A5‐aTN enables prolonged nanobody release during mucosal repair, allowing long‐term immune modulation. Future studies could use single‐cell transcriptomics to decipher functional shifts in immune subsets and explore interactions with microbiota metabolites to fully elucidate multilevel immunomodulatory mechanisms. Increased mucus secretion post‐treatment may provide ecological niches for commensals, amplifying immunomodulatory effects.

Compared to conventional small‐molecule drugs such as 5‐aminosalicylic acid, which rely on passive diffusion and non‐specific anti‐inflammatory mechanisms, our engineered EcN*Δlpp*::A5‐aTN strain offers markedly enhanced targeting and sustained therapeutic effects. The ANXA5 surface display enables active binding to PS exposed on inflamed intestinal tissues, promoting localized colonization and site‐specific delivery. Furthermore, the strain acts as an endogenous production system, continuously secreting anti‐TNF‐α nanobodies that achieve high‐affinity neutralization of TNF‐α through specific antigen‐antibody interactions. This mechanism ensures prolonged and precise therapeutic action within the inflammatory microenvironment, overcoming the transient distribution and lack of specificity characteristic of traditional small‐molecule agents.

Beyond advantages over conventional drugs, our engineered probiotic system also improves upon existing bacterial‐based therapeutic strategies in several key aspects: First, unlike approaches that use nanoparticle encapsulation to enhance colonization—a method susceptible to payload dilution upon bacterial replication—our strategy depends solely on stable genetic modifications for both targeting and therapeutic secretion, ensuring consistent production without loss of function. Second, the use of an anti‐TNF‐α nanobody not only offers superior biophysical properties such as small size and stability compared to conventional antibodies, but its secretion is further enhanced by the lpp knockout, which significantly improves protein release without compromising viability. Lastly, the ANXA5‐mediated targeting mechanism leverages a fundamental pathological feature—phosphatidylserine externalization—enabling universal applicability to inflamed tissues and positioning our platform as a versatile vehicle for future targeted biologics delivery.

This study has several limitations. First, EcN*Δlpp*::A5‐aTN showed excellent efficacy through localized anti‐TNF‐α nanobody delivery; however, EcN itself exhibits anti‐colitic effects that require further exploration. Second, efficacy was primarily evaluated in acute ulcerative colitis models. The effects of intervention in Crohn's disease models remain unknown and warrant investigation. Third, we observed that under optimally selected administration routes and dosage conditions, EcN*Δlpp*::A5‐aTN exhibited superior anti‐colitis effects compared to Infliximab. It should be noted, however, that the probiotic was administered orally (by gavage), while Infliximab was delivered via intraperitoneal injection—a distinction dictated by the fundamental differences between biological and live‐biotherapeutic agents.

In summary, we constructed an engineered inflammation‐targeting probiotic EcN*Δlpp*::A5‐aTN with enhanced colonization and high efficacy of anti‐TNF‐α nanobody secretion, and effectively alleviated colitis in mice. EcN*Δlpp*::A5‐aTN overcomes the limitations of systemic biologics and exhibits excellent safety, offering a promising therapeutic paradigm for precision intervention in IBD.

## 4 Experimental Section

### Reagents and Antibodies

The nuclear dye 4′,6‐diamidino‐2‐phenylindole (DAPI) and the membrane dye 3,3′‐dioctadecyloxacarbocyanine perchlorate (Dio) were purchased from Beyotime (Nantong, China). Dextran sulfate sodium (DSS, MW 36–50 kDa) was obtained from Yeasen Biotechnology (Shanghai, China). Mouse TNF‐α was obtained from Abclonal (Wuhan, China). Cycloheximide (CHX) was obtained from MedChemExpress (Monmouth Junction, NJ, USA). The cell‐permeable ROS probe 2′,7′‐Dichlorodihydrofluorescein diacetate (DCFH‐DA) and collagenase I was purchased from Sigma–Aldrich (St. Louis, Missouri, USA). Anti‐ANXA5 were procured from proteintech (Wuhan, China), Anti‐ZO‐1, occludin, myeloperoxidase (MPO), TNF‐α, and β‐Tubulin antibodies were procured from HuaAn Biotechnology (Hangzhou, China). Anti‐Myc, caspase‐8, cleaved‐caspase‐8, caspase‐3, cleaved‐caspase‐3, p‐p38, and p‐JNK antibodies were obtained from Cell Signaling Technology (Danvers, MA, USA). Fluorescently labeled antibodies, including anti‐CD45‐FITC, anti‐CD11b‐PE, anti‐F4/80‐BV421, anti‐Ly6G‐BV605, and anti‐Ly6C‐PE‐Cy7, were purchased from BD Biosciences (San Jose, CA).

### Bacterial Strains, Cell Lines, and Animals

To generate the engineered bacterial strains, expression plasmids carrying RFP, ANXA5, LuxCDABE, or aTN were constructed. All plasmids were based on a modified pET28a backbone containing a strong constitutive promoter. For periplasmic localization of ANXA5, an N‐terminal pelB signal sequence was incorporated. aTN were C‐terminally fused with a Myc tag to facilitate subsequent detection and quantification of protein expression. The resulting plasmids were individually transformed into EcN or the derived mutant EcN*Δlpp*::A5 via electroporation. Prior to electroporation, bacterial cultures were grown in LB to mid‐logarithmic phase, harvested, and washed three times with ice‐cold 10% glycerol to prepare electrocompetent cells. For each transformation, ≈100 ng of plasmid DNA was mixed with 100 µL of competent cells and electroporated at 2.5 kV, 25 µF, and 200 Ω. Immediately following electroporation, cells were recovered in 1 mL of LB medium for 1 h at 37 °C with shaking at 220 rpm, and then plated onto LB agar plates supplemented with 30 µg mL^−1^ kanamycin for selection. After overnight incubation at 37 °C, positive clones were selected and verified by colony PCR using the primers F1 (5′‐AGATCTCGATCCCGCGAAAT‐3′) and R1 (5′‐CAAGACCCGTTTAGAGGCCC‐3′). The following strains were successfully constructed and confirmed: EcN‐RFP, EcN‐ANXA5, EcN‐pelB‐ANXA5, EcN*Δlpp*::A5‐RFP, EcN‐LuxCDABE, EcN*Δlpp*::A5‐LuxCDABE, EcN‐aTN, EcN*Δlpp*::A5‐aTN.

CT26 mouse colon epithelial cells were cultured in DMEM supplemented with 10% FBS, 100 µg mL^−1^ streptomycin, and 100 U mL^−1^ penicillin at 37 °C with 5% CO_2_. All animal experiments were approved by the Animal Ethics Committee of China Pharmaceutical University (Approval No. 2024‐02‐011). Female C57BL/6 mice (8 weeks old, weighing 18–20 g) from Nanjing Annokang Biotechnology Co., Ltd. (Nanjing, China), were housed under specific pathogen‐free conditions (24 °C, 50% relative humidity) with ad libitum access to food and water for one week prior to the experiments.

### ANXA5‐RFP Intestinal Accumulation

Purified ANXA5‐RFP (20 mg kg^−1^) was injected intravenously via the tail vein into healthy and colitis‐prone mice. Thirty minutes later, the mice were euthanized and their colon and duodenum tissues were quickly collected. The tissues were fixed in 4% paraformaldehyde for 12 h, dehydrated in a 30% sucrose solution, and cryosectioned into 10 µm slices for observation with fluorescence microscopy.

### Deletion of *lpp* and Insertion of ANXA5 in the EcN Genome

The pCas9/pTargetT dual‐plasmid system to genetically modify the EcN genome was used.^[^ ^54^ ^]^ First, the pTargetT editing plasmid for *lpp* knockout and *ANXA*5 insertion was designed. This plasmid contained the sgRNA sequence (5′‐TAACCGTCGCTGGACAA‐3′) that targeted *lpp* for knockout. ANXA5 was also fused with an OmpA fragment (A46‐A159). The OmpA transmembrane domain anchored the fusion protein to the outer membrane, thereby exposing ANXA5 on the bacterial surface. EcN‐pCas9 cells were cultured in LB medium until the OD600 reached 0.2. Then, the cells were induced with 10 mM L‐arabinose to express the Cas9 protein. After further incubation until the OD600 reached 0.7, the cells were harvested to prepare competent EcN‐pCas9 cells. Then, 100 ng of the pTargetT plasmid was electroporated into the competent cells. Following transformation, the cells were recovered at 30 °C for 1 h and plated on LB agar supplemented with 30 µg mL^−1^ kanamycin and 50 µg mL^−1^ ampicillin. After an overnight incubation at 30 °C, the transformants were verified by PCR and DNA sequencing.

### Determination of Bacterial Growth Curve

Bacteria were inoculated into an LB medium and shaken at 37 °C and 220 rpm until the logarithmic growth phase was reached. The bacterial suspensions were then diluted to an OD_600_ of 0.05 with LB medium. Then, 200 µL of the suspension was added to each well of a 96‐well plate, with three replicate wells per group. After shaking every 30 min, the OD_600_ was measured for 20 h to monitor bacterial growth.

### Immunofluorescence Detection of ANXA5 Display on EcN Surface

Log‐phase bacteria were washed with PBS, blocked with 5% BSA for 1 h, and then incubated with the primary anti‐ANXA5 antibody overnight at 4 °C. The sample was then incubated with a fluorescent secondary antibody for 1 h in the dark and stained with DAPI for five minutes. ANXA5 expression on bacterial surfaces was observed by fluorescence microscopy and flow cytometry.

### In Vitro Evaluation of Bacterial‐Epithelial Cell Binding Capacity

CT26 cells were treated with 25 µg mL^−1^ CHX and 20 ng mL^−1^ TNF‐α for 24 h to induce apoptosis. Then, the cells were co‐cultured with EcN‐RFP or EcN*Δlpp*::A5‐RFP (MOI = 10:1) for 1 h. After washing with PBS to remove non‐specifically bound bacteria, flow cytometry was used to quantify the number of RFP‐positive bacteria that bound to the membrane of CT26 cells.

### Evaluation of Bacterial Colonization in Intestinal Tissue

For luminescent tracking, the mice received 1 × 10⁹ CFU of EcN‐LuxCDABE or EcN*Δlpp*::A5‐LuxCDABE. In vivo distribution was assessed at 6, 12, 24, 48, and 72 h using in vivo imaging system (Biolight Biotechnology Co., Ltd., Guangzhou, China). Mice were gavaged with 1 × 10⁹ CFU of either EcN‐RFP or EcN*Δlpp*::A5‐RFP, from 12 to 120 h post‐administration, the mice were euthanized and their colon tissues were homogenized. bacterial colonization was then compared using dilution plating. For histological analysis, the colon tissues of mice that were gavaged with 1 × 10⁹ CFU EcN‐RFP or EcN*Δlpp*::A5‐RFP were collected at the same time points. The tissues were then fixed in 4% paraformaldehyde at 4 °C for 4 h, washed with PBS, dehydrated in 30% sucrose at 4 °C overnight, embedded in optimal cutting temperature compound, cryosectioned at 10 µm, DAPI‐stained, and examined by fluorescence microscopy.

### In Vivo Safety Evaluation of Engineered Bacteria

Healthy mice received an oral gavage of 1 × 10⁹ CFU EcN*Δlpp*::A5 or EcN*Δlpp*::A5‐aTN daily for seven consecutive days. At the end of the study, the morphology of the heart, liver, spleen, lungs, and kidneys was examined, and organ indices (organ weight/body weight×100) were calculated. Serum was collected to assess liver function by measuring alanine aminotransferase (ALT) and aspartate aminotransferase (AST), and to evaluate kidney function by measuring blood urea nitrogen (BUN) and creatinine (CREA). The organs were fixed in 4% paraformaldehyde, paraffin‐embedded, sectioned, and stained with hematoxylin and eosin (H&E) for histopathological examination under an optical microscope.

### DSS‐Induced Ulcerative Colitis Model, Intervention, and Evaluation

A 3.5% DSS solution was prepared in sterile water and filtered through a 0.22 µm membrane. Model groups received 3.5% DSS as their sole drinking water, while control mice drank sterile water. The intervention groups received a daily oral gavage of 1 × 10⁹ CFU of wild‐type EcN, EcN*Δlpp*::A5, or EcN*Δlpp*::A5‐aTN during the exposure to DSS. The positive control group received intraperitoneal infliximab injections at 10 mg kg^−1^ on days 3 and 5.

Disease activity was scored based on three parameters: weight loss percentage (1: 1%–5%; 2: 5%–10%; 3: 10%–20%; 4: >20%), stool consistency (0: normal; 1: soft; 2: mucoid; 3: liquid), and fecal bleeding (0: none; 1: Hemoccult positive; 2: Hemoccult positive and visual pellet bleeding; 4: gross bleeding, blood around anus).^[^ ^55^ ^]^ DAI score was the sum of these subscores. Colon tissue damage was evaluated using H&E staining to assess inflammatory cell infiltration, epithelial structural destruction, and crypt damage. Mucin secretion was detected by Alcian Blue staining, and epithelial cell proliferation was evaluated by Ki67 staining.

### Immunofluorescence

The mouse colon tissues were fixed in 4% paraformaldehyde at 4 °C for 24 h. Then, they were dehydrated through graded ethanol, cleared in xylene, paraffin‐embedded, and sectioned at 5 µm. After deparaffinization and rehydration, antigen retrieval was performed in a citrate buffer solution using microwave heating for 15 min. The sections were blocked with 10% goat serum at room temperature for 30 min. Then, they were incubated with the primary antibody overnight at 4 °C. After three washes with PBS, the sections were incubated with the FITC‐conjugated secondary antibody for 50 min at room temperature. After an additional wash with PBS, the nuclei were stained with DAPI. The sections were mounted with an antifade mounting medium and observed under a fluorescence microscope.

### Immunohistochemistry

The tissue was processed as described in above section. Sections were incubated with the primary antibody overnight at 4 °C, washed with PBS, incubated with the secondary antibody for 50 min at room temperature, washed again, and developed with the DAB substrate. Then, counterstaining was performed with hematoxylin and eosin. Next, the sections were dehydrated through graded ethanol, cleared in xylene, and mounted with neutral resin for microscopic observation.

### Mouse Colonic Epithelial Cell Isolation and ROS Detection

After euthanizing the mice, their colons were rinsed thoroughly with ice‐cold PBS and the fat tissues and Peyer's patches were removed. The tissues were opened longitudinally, cut into 0.5 cm^2^ pieces, and digested in RPMI 1640 medium containing 3% serum, 2 mM EDTA, and 1 mM DTT. The digestion occurred at 37 °C with shaking for 40 min, with the mixture being vortexed every 20 min to promote dissociation. The resulting suspension was filtered through a 200‐mesh sieve and centrifuged at 400×g for five minutes at 4 °C to collect the epithelial cells. After washing twice with serum‐free medium, the cells were incubated with a 10 µM DCFH‐DA probe at 37 °C in the dark for 30 min. After removing the unbound probe by washing, the relative ROS levels were analyzed by flow cytometry (excitation at 488 nm and detection at 530 ± 15 nm).

### Mouse Lamina Propria Immune Cell Isolation and Flow Cytometry

After the removal of epithelial cells, as described in above section, the remaining colon tissues were washed once with ice‐cold PBS. The tissues were then minced and digested in RPMI 1640 containing 1 mg mL^−1^ collagenase I at 37 °C with shaking for 80 min. The digestion process was interrupted by vortexing every 20 min. Digestion was terminated on ice and the suspension was filtered twice through a 200‐mesh cell strainer. The cells were collected by centrifugation at 400×g for 10 min at 4 °C. Then, the cells were resuspended in 44% Percoll and layered onto 67% Percoll. The cells were then centrifuged at 800×g for 20 min at room temperature. The cells at the interface were collected, washed three times with PBS, and resuspended to obtain lamina propria immune cells. After blocking Fc receptors, the cells were stained with anti‐CD45‐FITC, anti‐CD11b‐PE, anti‐F4/80‐BV421, anti‐Ly6G‐BV605, and anti‐Ly6C‐PE‐Cy7 antibodies at 4 °C in the dark for 40 min. After washing, immune cell populations were analyzed by flow cytometry. First, CD45^+^ cells were gated to identify total immune cells. Then, the proportion of F4/80^+^ macrophages was analyzed. Next, CD45^+^CD11b^+^ cells were gated to analyze the proportions of Ly6G^+^ neutrophils and Ly6C^+^ monocytes.

### mRNA Extraction and qPCR Detection

Total RNA was extracted from mouse colon tissues using TRIzol reagent. Approximately 50 mg of minced intestinal tissue was homogenized in 1 mL TRIzol reagent at 4 °C. The homogenate was mixed with 200 µL chloroform, vigorously shaken for 15 s, incubated for 5 min, and centrifuged at 12 000 rpm for 15 min. The upper aqueous phase containing RNA was transferred, mixed with an equal volume of isopropanol, inverted several times, and incubated on ice for 10 min. After centrifugation at 12 000 rpm for 10 min at 4 °C, the supernatant was discarded, and the pellet was washed with 1 mL 75% ethanol before dissolving in RNase‐free water. cDNA was synthesized from 1 µg total RNA using HiFiScript gDNA Removal RT MasterMix (Cowin Biotech, Taizhou, China). qPCR was performed using ChamQ SYBR qPCR Master Mix (Vazyme, Nanjing, China) with SYBR Green detection. β‐Actin served as the reference gene, and relative quantification was performed using the 2^−ΔΔCt^ method. Primer sequences were as follows: IL‐1β F: 5′‐GCAACTGTTCCTGAACTCAACT‐3′, IL‐1β R: 5′‐ATCTTTTGGGGTCCGTCAACT‐3′; TNF‐α F: 5′‐CCCTCACACTCAGATCATCTTCT‐3′, TNF‐α R: 5′‐GCTACGACGTGGGCTACAG‐3′; IL‐6 F: 5′‐TAGTCCTTCCTACCCCAATTTCC‐3′, IL‐6 R: 5′‐TTGGTCCTTAGCCACTCCTTC‐3′; IL‐17A F: 5′‐TTTAACTCCCTTGGCGCAAAA‐3′, IL‐17A R: 5′‐CTTTCCCTCCGCATTGACAC‐3′; β‐Actin F: 5′‐GGCTGTATTCCCCTCCATCG‐3′, β‐Actin R: 5′‐CCAGTTGGTAACAATGCCATGT‐3′.

### Western Blot

Fresh mouse colon tissue was snap‐frozen in liquid nitrogen and homogenized in RIPA lysis buffer containing protease inhibitors. After 30 min of sonication and lysis, the lysates were centrifuged at 12 000 rpm for 15 min at 4 °C, and the resulting supernatants were collected. Protein concentrations were quantified by the BCA assay. The samples were mixed with loading buffer, denatured at 100 °C for 5 min, and separated by 10% SDS‐PAGE (80 V stacking and 120 V separation). Then, they were transferred to PVDF membranes via wet transfer (300 mA for 90 min). The membranes were blocked with 5% non‐fat milk at room temperature for 1 h, incubated with the primary antibody overnight at 4 °C, washed three times with TBST, incubated with the HRP‐conjugated secondary antibody at room temperature for 1 h, washed again, and developed using electrogenerated chemiluminescence (GlpBio, Montclair, CA, USA).

### 16S rRNA Sequencing

Fecal samples from C57BL/6 mice were flash‐frozen in liquid nitrogen and stored at −80 °C. Total microbial DNA was extracted by Shanghai OE Biotech Co., Ltd (Shanghai, China). The V3‐V4 region of the 16S rRNA gene was amplified via PCR, followed by paired‐end sequencing.

### Statistical Analysis

Statistical analyses were conducted using GraphPad Prism software (version 10.1.2, La Jolla, USA), with continuous data expressed as mean ± SD. Based on data characteristics, the appropriate statistical test was applied: unpaired two‐tailed Student's *t*‐test for comparisons between two groups; one‐way ANOVA followed by Tukey's post hoc test for comparisons among ≥3 groups; or two‐way ANOVA followed by Sidak's post hoc test for datasets involving two independent variables. A p‐value < 0.05 was considered statistically significant.

## Conflict of Interest

The authors declare no conflict of interest.

## Author Contributions

S.H. performed conceptualization, methodology, investigation, formal analysis, visualization, and wrote the original draft. K.L. performed investigation, formal analysis, and validation. P.S. performed investigation, and formal analysis. L.L. performed methodology, and visualization. J.P. performed investigation. B.Z. performed conceptualization, methodology, investigation, formal analysis, wrote‐reviewed and edited. Z.H. performed conceptualization, wrote‐reviewed and edited, supervision, and funding acquisition.

## Supporting information



Supporting Information

## Data Availability

The data that support the findings of this study are available from the corresponding author upon reasonable request.
