# Supplementary material for: An Inflammation‐Targeting Engineered Probiotic Escherichia coli Nissle 1917 with High Anti‐TNF‐α Nanobody Secretion Efficacy Alleviates Ulcerative Colitis
Source: Adv Sci (Weinh). 2025 Sep 29;12(46):e12360. doi: 10.1002/advs.202512360 (PMC12697777; doi:10.1002/advs.202512360)
Supplement: Supplementary file 1 — Supporting Information [file ADVS-12-e12360-s001.docx]

Supporting Information

**An Inflammation-Targeting Engineered Probiotic *Escherichia coli* Nissle 1917 with High anti-TNF-α Nanobody Secretion Efficacy Alleviates Ulcerative Colitis**

*Siqi Hua, Kaiqiang Li, Pengyou Shang, Lina Liu, Jiayu Pan, Bo Zhu^*^, Zichun Hua^*^*

**Supplementary Method**

**Details of the Fusion Between ANXA5 and the OmpA Fragment**

In our study, we engineered EcN to display ANXA5 on its outer membrane. This was achieved by fusing the ANXA5 coding sequence to the C-terminus of an OmpA fragment (A46-A159), which was preceded by a signal peptide. The resulting fusion protein, Signal Peptide-OmpA-ANXA5, is secreted into the periplasm where the OmpA domain anchors it to the outer membrane, effectively presenting ANXA5 on the cell surface. The detailed genetic construct is as follows: the fusion begins with the signal peptide (green), followed by the OmpA fragment (purple), a linker (black), and finally the ANXA5 gene sequence (blue).

5'-AgaaagctactaaactggtactgggcgcggtaatcctgggttctactctgctggcaggttgctccagcaacgctaaaatcgatcagggtatcCCGTATGTTGGCTTTGAAATGGGTTACGACTGGTTAGGTCGTATGCCGTACAAAGGCGACAACATCAACGGCGCATACAAAGCTCAGGGCGTTCAGCTGACCGCTAAACTGGGTTACCCAATCACTGACGATCTGGACATCTACACTCGTCTGGGTGGTATGGTATGGCGTGCAGACACCAAGGCTAACGTACCTGGTGGCGCATCCTTTAAAGACCACGACACCGGCGTTTCTCCGGTCTTCGCTGGCGGTGTTGAGTATGCGATCACTCCTGAAATCGCTACCCGTCTGGAATACCAGTGGACCAACAACATCGGTGACGCACACACCATCGGCACTCGTCCGGACAACggtatcccgtccagcggtATGGCACAGGTTCTCAGAGGCACTGTGACTGACTTCCCTGGATTTGATGAGCGGGCTGATGCAGAAACTCTTCGGAAGGCTATGAAAGGCTTGGGCACAGATGAGGAGAGCATCCTGACTCTGTTGACATCCCGAAGTAATGCTCAGCGCCAGGAAATCTCTGCAGCTTTTAAGACTCTGTTTGGCAGGGATCTTCTGGATGACCTGAAATCAGAACTAACTGGAAAATTTGAAAAATTAATTGTGGCTCTGATGAAACCCTCTCGGCTTTATGATGCTTATGAACTGAAACATGCCTTGAAGGGAGCTGGAACAAATGAAAAAGTACTGACAGAAATTATTGCTTCAAGGACACCTGAAGAACTGAGAGCCATCAAACAAGTTTATGAAGAAGAATATGGCTCAAGCCTGGAAGATGACGTGGTGGGGGACACTTCAGGGTACTACCAGCGGATGTTGGTGGTTCTCCTTCAGGCTAACAGAGACCCTGATGCTGGAATTGATGAAGCTCAAGTTGAACAAGATGCTCAGGCTTTATTTCAGGCTGGAGAACTTAAATGGGGGACAGATGAAGAAAAGTTTATCACCATCTTTGGAACACGAAGTGTGTCTCATTTGAGAAAGGTGTTTGACAAGTACATGACTATATCAGGATTTCAAATTGAGGAAACCATTGACCGCGAGACTTCTGGCAATTTAGAGCAACTACTCCTTGCTGTTGTGAAATCTATTCGAAGTATACCTGCCTACCTTGCAGAGACCCTCTATTATGCTATGAAGGGAGCTGGGACAGATGATCATACCCTCATCAGAGTCATGGTTTCCAGGAGTGAGATTGATCTGTTTAACATCAGGAAGGAGTTTAGGAAGAATTTTGCCACCTCTCTTTATTCCATGATTAAGGGAGATACATCTGGGGACTATAAGAAAGCTCTTCTGCTGCTCTGTGGAGAAGATGAC-3'

**The Gene Sequence for Red Fluorescent Protein**

The gene sequence for red fluorescent protein is as follows:

5'-ATGGTGTCTAAGGGCGAAGAGCTGATTAAGGAGAACATGCACATGAAGCTGTACATGGAGGGCACCGTGAACAACCACCACTTCAAGTGCACATCCGAGGGCGAAGGCAAGCCCTACGAGGGCACCCAGACCATGAGAATCAAGGTGGTCGAGGGCGGCCCTCTCCCCTTCGCCTTCGACATCCTGGCTACCAGCTTCATGTACGGCAGCAGAACCTTCATCAACCACACCCAGGGCATCCCCGACTTCTTTAAGCAGTCCTTCCCTGAGGGCTTCACATGGGAGAGAGTCACCACATACGAAGACGGGGGCGTGCTGACCGCTACCCAGGACACCAGCCTCCAGGACGGCTGCCTCATCTACAACGTCAAGATCAGAGGGGTGAACTTCCCATCCAACGGCCCTGTGATGCAGAAGAAAACACTCGGCTGGGAGGCCAACACCGAGATGCTGTACCCCGCTGACGGCGGCCTGGAAGGCAGAAGCGACATGGCCCTGAAGCTCGTGGGCGGGGGCCACCTGATCTGCAACTTCAAGACCACATACAGATCCAAGAAACCCGCTAAGAACCTCAAGATGCCCGGCGTCTACTATGTGGACCACAGACTGGAAAGAATCAAGGAGGCCGACAAAGAGACCTACGTCGAGCAGCACGAGGTGGCTGTGGCCAGATACTGCGACCTCCCTAGCAAACTGGGGCACAAACTTAAT-3'

**The Gene Sequence for Anti-TNF-α Nanobody**

The gene sequence for anti-TNF-α nanobody is as follows:

5'-ATGGATGTTCAGCTGGTTGAAAGCGGTGGTGGTTCTGTTCAGGCGGGTGGTAGCCTGCGTCTGAGCTGTGCTGCTTCTGGTTATACTAGCTCTTCTTGTTCTATGGGTTGGTACCGTCAGGCGCCGGGTAAAGAACGTGAACTGGTTGCGACCATCTTTGCTGATGGTCGTACCCGTTATGCGGATAGCGTTAAAGGTCGTTTCACCATCTCTCGTGATAACGCGAAAAACACCGTTTATCTGCAGATGAACAGCCTGAAACCGGAAGATACCGCTATGTACTACTGCAACACCGATCCGCTGCGTAGCTACTCTGATTATGATTGCGTTAACTGGAACAACTATTGGGGTCAGGGTACCCAGGTTACCGTTTCTTCT-3'

**LuxCDABE Sequence**

The sequance for LuxCDABE was cloned from pMM643 (Addgene Plasmid #112533).

**Supplementary Figures**


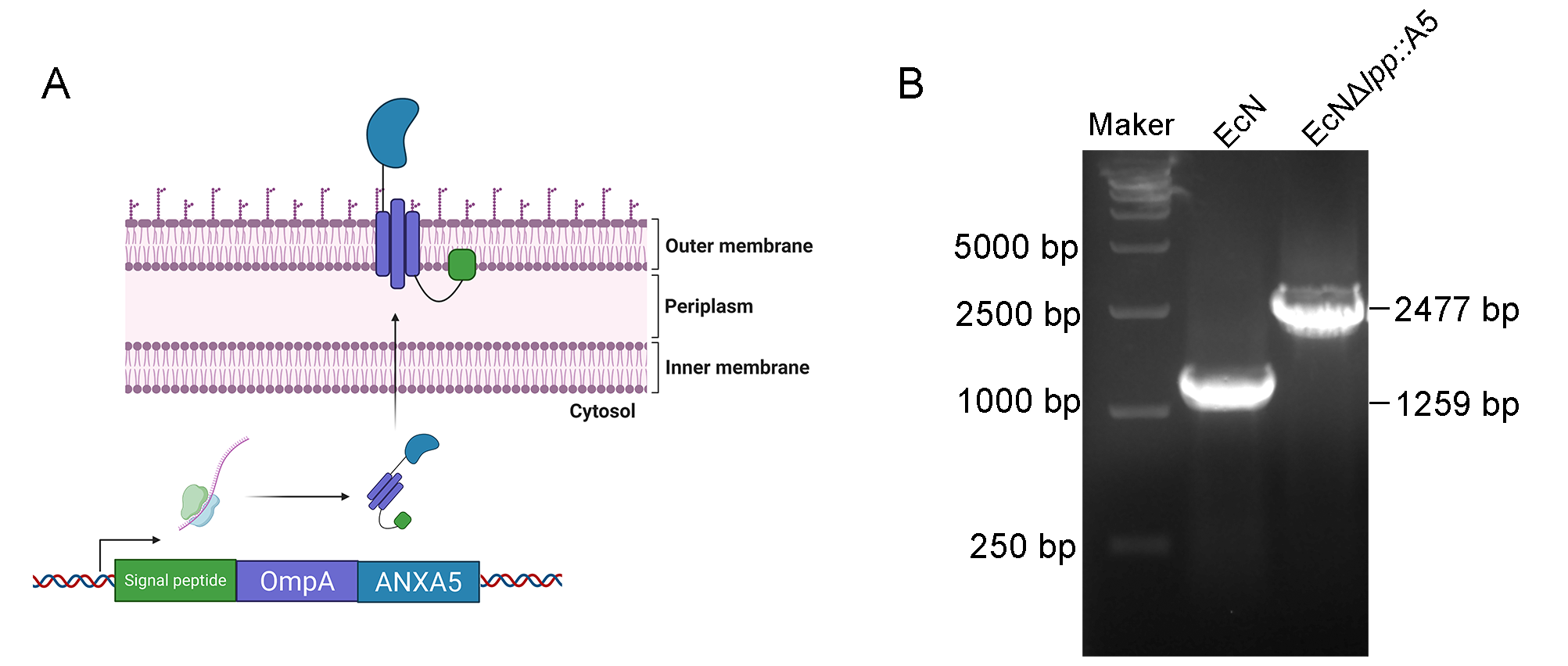


**Figure S1. Schematic representation and PCR verification of ANXA5 display on the surface of EcN*△lpp*::A5.** (A) A schematic diagram illustrating the display of ANXA5 on the surface of engineered EcN. The ANXA5 coding sequence was fused to the C-terminus of a signal peptide and OmpA fragment. The resulting fusion protein, Signal Peptide-OmpA-ANXA5, is secreted into the periplasm, where the OmpA domain anchors it to the outer membrane, effectively presenting ANXA5 on the bacterium surface. (B) Using specific primers (F: 5'-CAGTTAGTACTGAGCAAAGGTGTTGTG-3'; R: 5'-TCGCGCATGGTAATGGCGAATACCTC-3') designed based on the insertion site flanking sequences, we performed PCR amplification on the genomic DNA of the recombinant strain containing the inserted Signal Peptide-OmpA-ANXA5. Analysis by 1% agarose gel electrophoresis showed a clear, specific amplification band at the expected size of 2477 bp, confirming that Signal Peptide-OmpA-ANXA5 was successfully integrated into the EcN genome.

**
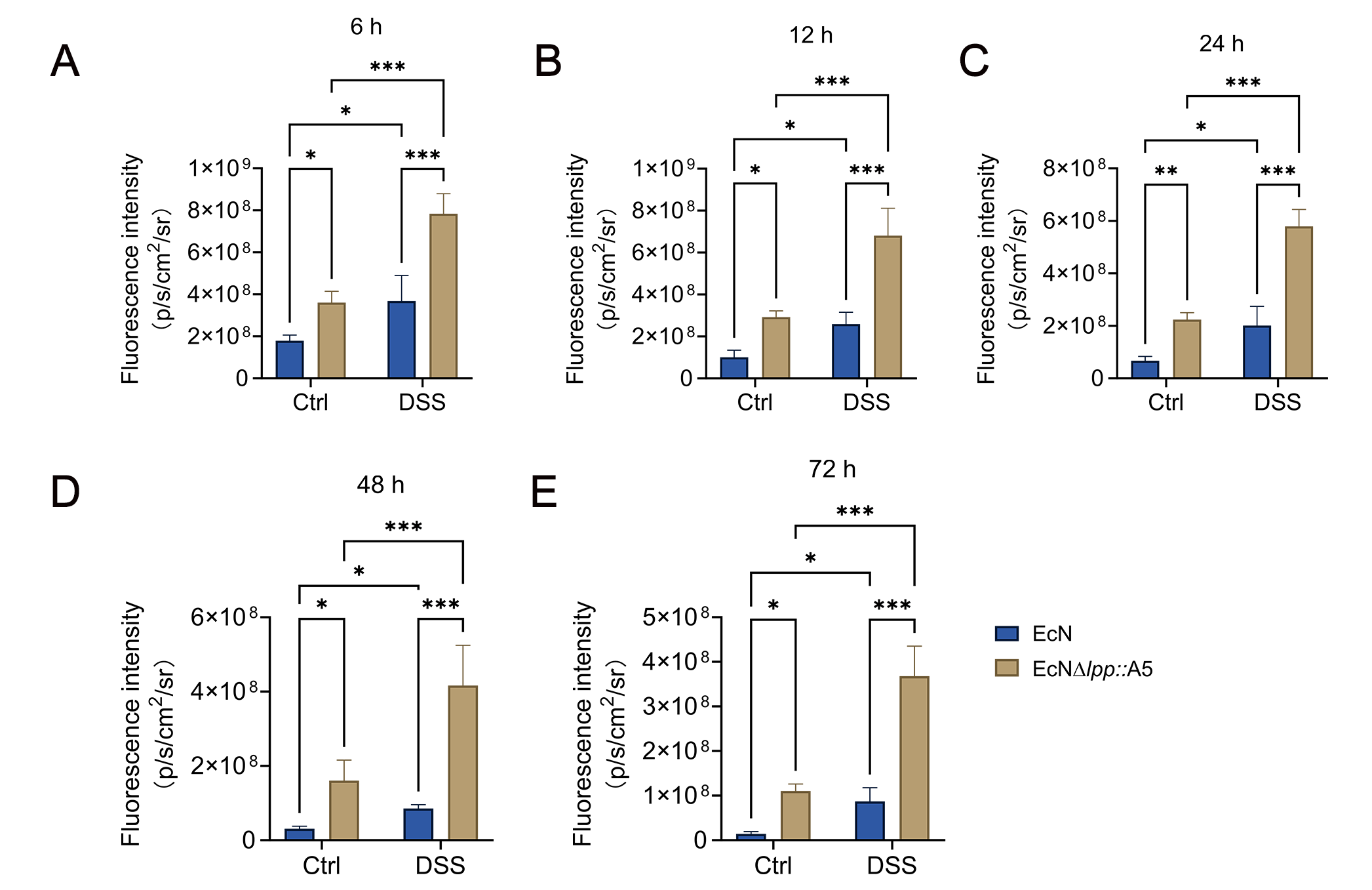
**

**Figure S2. Effect of inflammation on colon colonization by EcN*Δlpp*::A5 and EcN in mice.** The LuxCDABE bioluminescence system was introduced into EcN and EcN*Δlpp*::A5, respectively. DSS-induced colitis mice and control mice were orally gavaged with 1×10⁹ CFU of each bacterial strain. Colon colonization capacity was assessed using an *in vivo* imaging system at 6 (A), 12 (B), 24 (C), 48 (D), and 72 hours (E) after administration (n = 3). Statistical analysis was performed using two-way ANOVA followed by Sidak’s test (two independent variables). **P* < 0.05, ***P* < 0.01, ****P* < 0.001.

**
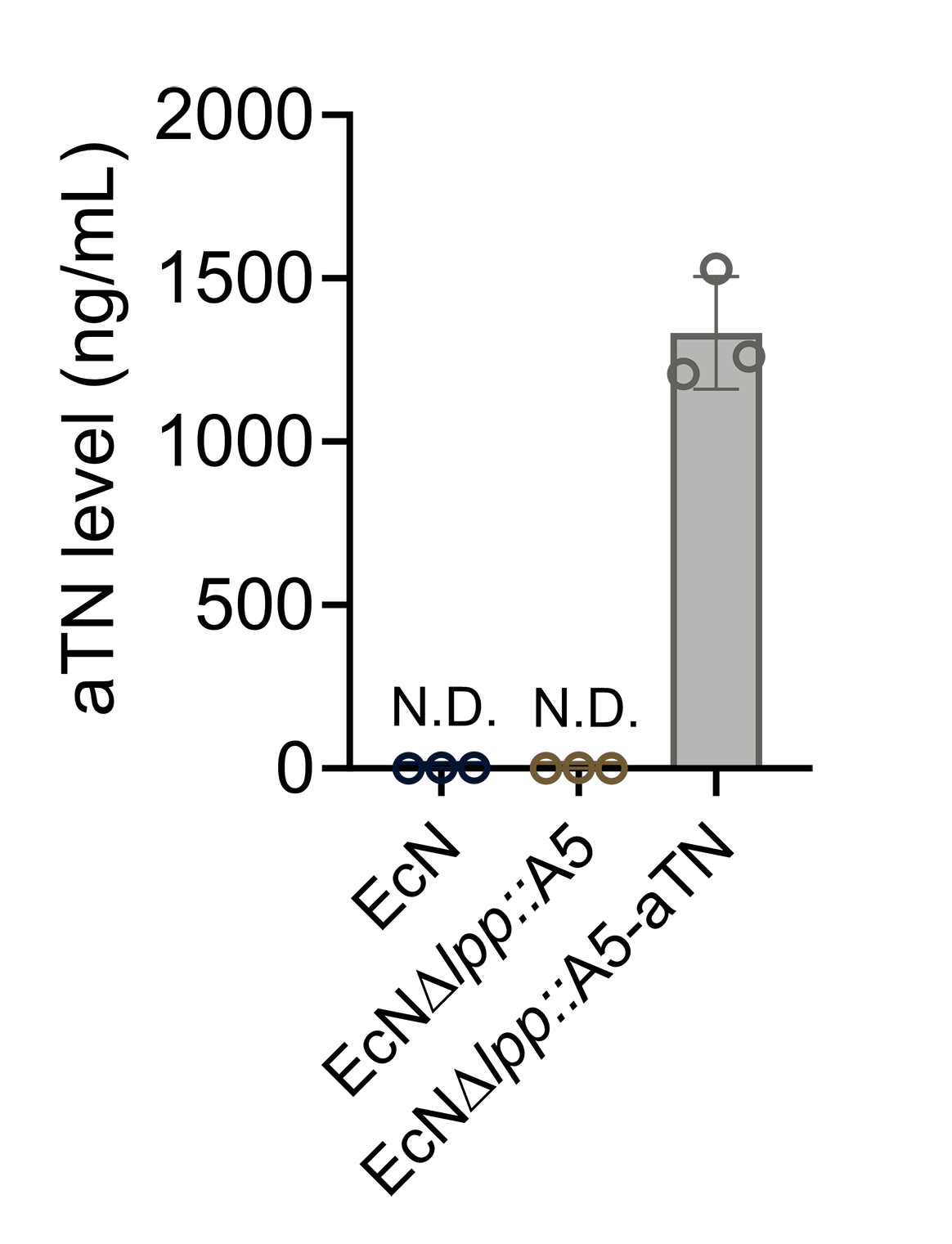
**

**Figure S3. Expression of aTN protein in EcN*Δlpp*::A5-aTN.** EcN*Δlpp*::A5-aTN were cultured in LB medium at 37°C with shaking at 220 rpm until the optical OD_600_ reached 0.9, corresponding to a bacterial concentration of approximately 1×10^9^ CFU/mL. The culture supernatant was then centrifuged at 8000×g for 10 minutes. To remove potential bacterial debris, the supernatant was filtered through a 0.22 μm filter. Since the aTN C-terminus carried a Myc-tag, the concentration of aTN in the resulting supernatant was then determined using a commercial Myc-tag ELISA kit. EcN*Δlpp*::A5 and wild-type EcN were used as negative controls. Based on the molecular weights of the Myc-tag and aTN, we calculated the aTN concentration in the supernatant to be 1332.79 ± 172.28 ng/mL. The results are based on three biological replicates and are presented as the mean ± SD. N.D.: not detected.


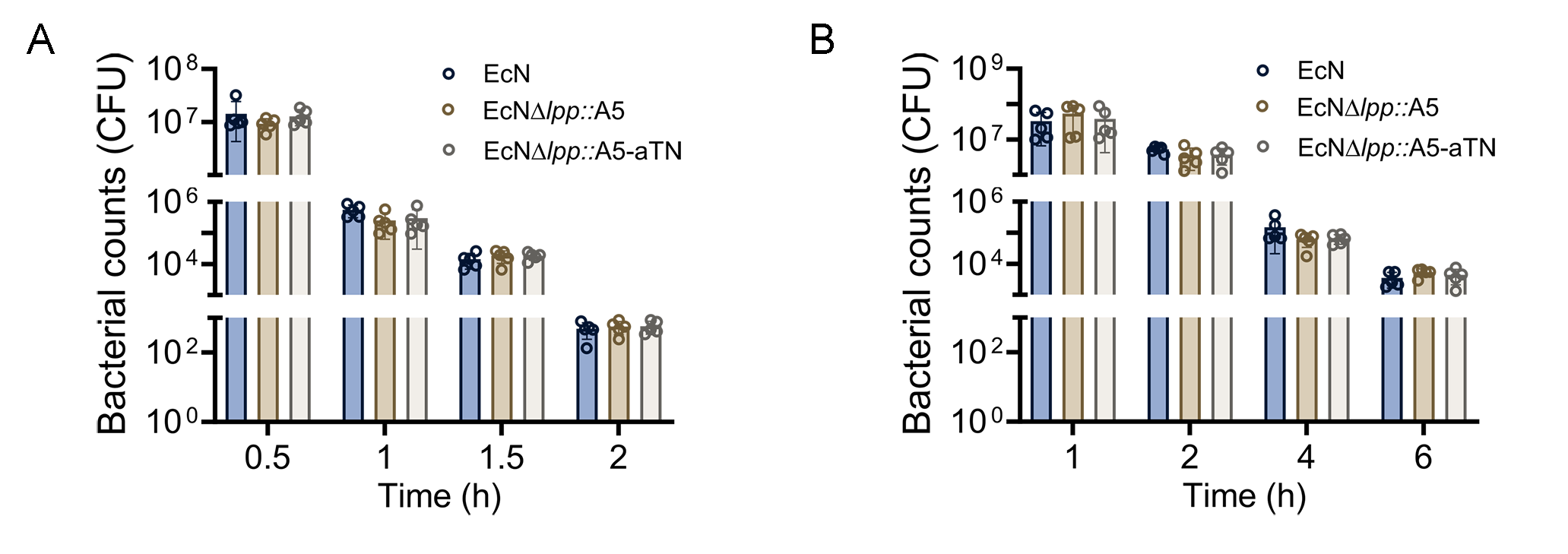


**Figure S4. *In vitro* gastrointestinal resistance of engineered bacteria.** To evaluate the bacterial tolerance in simulated gastrointestinal conditions, wild-type EcN and its engineered strains EcN*Δlpp*::A5 and EcN*Δlpp*::A5-aTN were inoculated into LB medium and cultured to the logarithmic growth phase at 37°C with 220 rpm shaking. Bacterial cells were collected, and equal amounts of bacterial suspensions (1×10⁹ CFU) were exposed to artificial simulated gastric fluid (A) and bile salts (B), respectively. Samples were taken at different time points, plated on LB agar plates, and incubated overnight at 37°C for colony counting to assess the survival rate of each strain (n=5). Statistical analyses were performed using two-way ANOVA with Sidak’s test (two independent variables). ns: not significant.


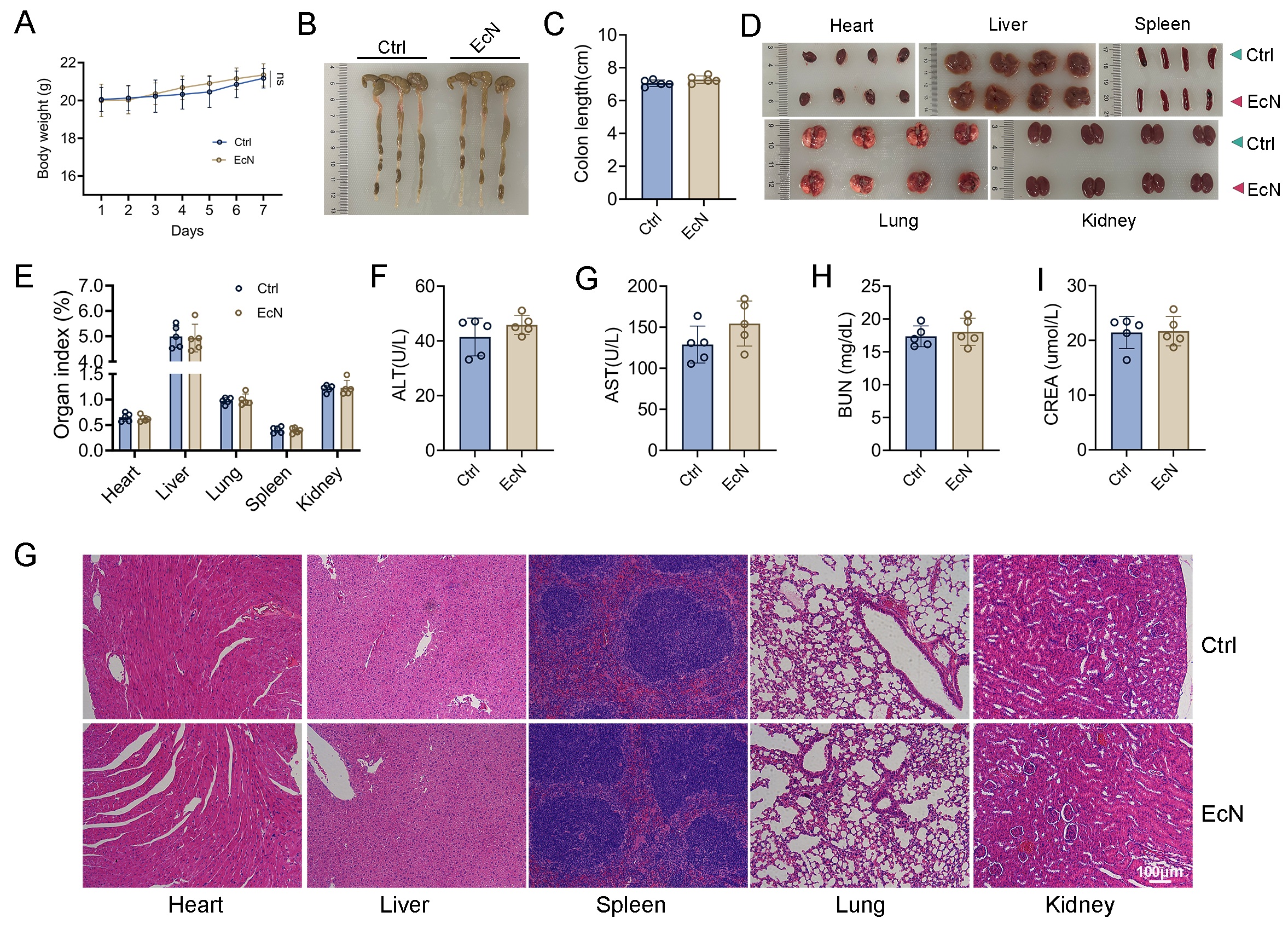


**Figure S5. Safety evaluation of EcN.** Healthy mice received an oral gavage of 1×10⁹ CFU of wild type EcN for seven days (n = 5). (A) Mouse body weight changes. (B–C) On day 7, the mice were euthanized, and the colon length was measured. (D) Heart, liver, spleen, lung and kidney tissues were photographed (n = 4). (E) Organ indexes were calculated as organ weight/body weight×100 (n = 5). (F–I) Mouse serum was isolated to detect liver injury markers (ALT and AST) and kidney injury markers (BUN and CREA, n = 5). (J) H&E staining was used to evaluate tissue damage in heart, liver, spleen, lung and kidney (n = 5; representative images are shown). Statistical analyses were performed using one-way ANOVA with Tukey’s test (≥3 groups), or two-way ANOVA with Sidak’s test (two independent variables). ns: not significant.


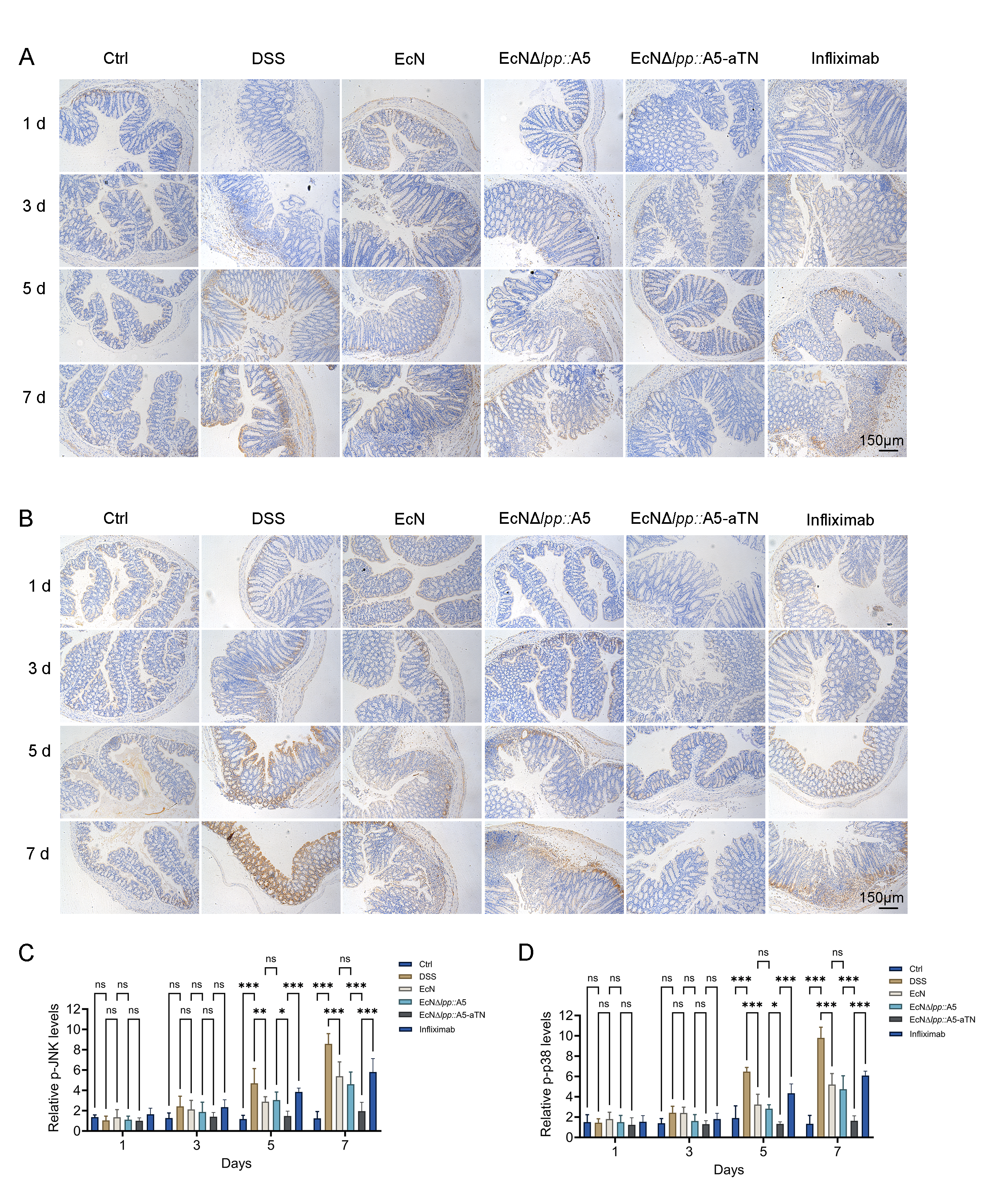


**Figure S6. Dynamic changes of colon p-JNK and p-p38 affected by EcN*Δlpp*::A5-aTN in DSS colitis mice.** The UC mouse model was established using 3.5% DSS as the sole source of drinking water. The treatment groups received an oral gavage of 1×10⁹ CFU of EcN, EcN*Δlpp*::A5-aTN , or EcN*Δlpp*::A5-aTN daily. DSS colitis mice that received an equal volume of PBS served as the model control group (n = 5). Non-modeled mice served as the healthy control group (n = 5). DSS-treated mice that received 10 mg/kg infliximab via intraperitoneal injection on days 3 and 5 served as the positive control group (n = 5). On treatment days 1, 3, 5, and 7, mice from each group were euthanized, and colon tissues were collected for immunohistochemical analysis to detect p-JNK (A, C) and p-p38 (B, D) levels. Representative images are shown in (A) and (B). Data are presented as mean ± SD. Statistical analyses were performed using two-way ANOVA with Sidak’s test (two independent variables). **P* < 0.05, ***P* < 0.01, ****P* < 0.001, ns: not significant.
